# Ligand-Induced Activation of Single-Atom Palladium Heterogeneous Catalysts for Cross-Coupling Reactions

**DOI:** 10.1021/acsnano.4c14131

**Published:** 2025-01-02

**Authors:** Dario Poier, Oliver Loveday, Marc Eduard Usteri, Dragos Stoian, Núria López, Sharon Mitchell, Roger Marti, Javier Pérez-Ramírez

**Affiliations:** † Institute of Chemical Technology, Haute École d’Ingénierie et d’Architecture Fribourg, HES-SO University of Applied Sciences and Arts Western Switzerland, 1700 Fribourg, Switzerland; ‡ Institute of Chemical Research of Catalonia (ICIQ-CERCA), The Barcelona Institute of Science and Technology (BIST), 43007 Tarragona, Spain; § Department of Physical and Inorganic Chemistry, Universitat Rovira i Virgili, 43007 Tarragona, Spain; ∥ Department of Chemistry and Applied Biosciences, Institute for Chemical and Bioengineering, ETH Zurich, 8093 Zurich, Switzerland; ⊥ Swiss Norwegian Beamlines, European Synchrotron Radiation Facility, 38043 Grenoble, France

**Keywords:** single-atom catalysis, cross-coupling reactions, Pd_1_@NC, ligand selection, phosphine, structure-performance relations, active-site accessibility

## Abstract

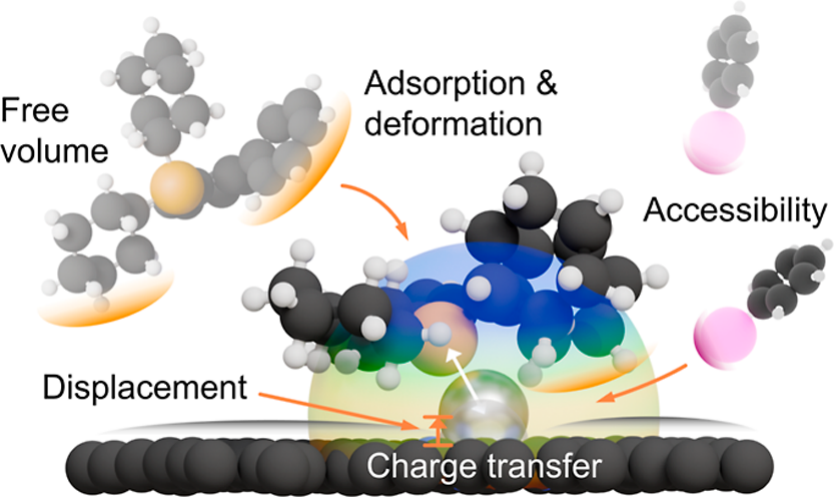

Single-atom heterogeneous catalysts (SACs) are potential, recoverable alternatives to soluble organometallic complexes for cross-coupling reactions in fine-chemical synthesis. When developing SACs for these applications, it is often expected that the need for ligands, which are essential for organometallic catalysts, can be bypassed. Contrary to that, ligands remain almost always required for palladium atoms stabilized on commonly used functionalized carbon and carbon nitride supports, as the catalysts otherwise show limited activity. Despite this, ligand optimization has received little attention, and their role in activating SACs is poorly understood. Here, we explore the impact of structurally diverse phosphine ligands on the performance of nitrogen-doped carbon supported single-atoms (Pd_1_@NC) in the Sonogashira–Hagihara (SH) cross-coupling reaction, using X-ray absorption spectroscopy and density functional theory simulations to rationalize the observed trends. Compared to the ligand-free SAC, SH activity is enhanced in almost all ligand-assisted systems, with reactivity varying by up to 8 orders of magnitude depending on the ligand choice. Distinct trends emerge based on the free ligand volume and ligand class. Unlike molecular systems, the electronic effects of phosphine ligands are less significant in SACs due to the modulating influence of the support. Instead, the performance of SAC-ligand systems is governed by a balance between the ligand deformation energy during coordination with metal centers, and their resulting accessibility to cross-coupling reagents. These findings offer key insights into optimizing Pd-SACs by leveraging phosphine ligands to activate metal centers and tailor the 3D environment.

Since the discovery of the catalytic potential of transition metals, they have become pivotal in global research and the manufacturing of many societally relevant chemical products.^1,2^ Transition-metal-catalyzed C–C and C–N bond-forming reactions like the Suzuki–Miyaura (SM, 2010 Nobel Prize in Chemistry), Buchwald–Hartwig (BH), or Sonogashira–Hagihara (SH) cross-couplings equip synthetic chemists with enormous versatility.^3−6^ However, the high costs and environmental impact associated with using molecular catalysts, particularly due to the challenges of fully recovering the precious metals often involved, are growing concerns.^7−9^

Heterogeneous single-atom catalysts (SACs), featuring spatially isolated, monoatomic active sites stabilized on suitable solid support materials, attract attention as potentially sustainable alternatives due to their recoverability, and thus the possibility to significantly reduce their environmental impact.^10,11^ The properties of palladium SACs in many respects resemble those of organometallic complexes, where the support material plays the role of ligands in stabilizing the isolated metal centers and determining the electronic properties.^12,13^ Palladium SACs, in particular, have demonstrated high selectivity and promising yields in various coupling applications, and the potential for full metal recovery.^14,15^

When applying SACs in simple cross-coupling reactions, ligands may seem unnecessary, as the support-metal interaction should be strong enough to stabilize the metal centers during the catalytic cycle. However, many studies on SACs still incorporate ligands, particularly when using common functionalized carbon and carbon nitride supports.^16,17^ Although efforts to develop ligand-free heterogeneous catalysts for SH coupling, such as carbon-coated metal oxide rods, have been reported, these systems required higher reaction temperatures, exceeding 383 K, to provide competitive results, and their stability remains unverified.^18^ Despite their beneficial effect on performance, little attention has been given to the role of ligands beyond identifying one that functions, usually phosphine-based, and optimizing its quantity. Recent research on Pd_1_@C_3_N_4_ SACs in SM coupling has suggested that triphenylphosphine might play an activating role, as evidenced by in situ XAS findings that showed a small modulation of the electronic properties of palladium due to the ligand interaction.^19^

In organometallic catalysis, modifying the coordination environment and electronic state of metal centers with ligands has been a key approach to enhance functionality.^20−22^ Comparatively, the ligand selection on SAC-phosphine systems remains unexplored, and there is a lack of understanding of whether ligand design principles from metal complexes can be transferred to SACs. Nonetheless, incorporating ligands could further boost SAC reactivity without compromising environmental benefits, as studies have shown that ligands have a minimal impact on the overall environmental footprint of SAC-catalyzed reactions.^23,24^

In this work, we elucidate the structure-reactivity relationships of Pd_1_@NC-ligand systems in the Sonogashira–Hagihara coupling, focusing on a diverse range of phosphines. Our evaluation shows that most ligands significantly enhance catalytic activity, which is negligible in their absence. The activity correlates with the average volume of the free ligand conformer, though distinct trends emerge. To rationalize these variations, we employ quasi-in situ X-ray absorption spectroscopy (XAS) and density functional theory (DFT) simulations to examine the geometric and electronic effects of different SAC-ligand systems. We demonstrate that activation stems from the displacement of palladium centers out from the metal coordination sites in the host, revealing a complex interplay between system properties and performance. The rules for ligand selection deviate from those traditionally applied to organometallic complexes. This study represents a pivotal step in SAC development, offering insights to improve their performance in various synthetic applications.

## Results and Discussion

### Phosphine Reactivity Trends

In 2022 some of us demonstrated the potential of SACs for SH coupling (Scheme S1), showing that palladium atoms anchored on nitrogen-doped carbon (Pd_1_@NC) could achieve relevant yields without metal loss.^10^ A lifecycle assessment comparing the environmental benefits of Pd_1_@NC to those of a typical homogeneous catalyst yielded two key conclusions; first, the ability to fully recover palladium could reduce the environmental footprint by orders of magnitude; second, the contribution of the phosphine ligand was almost negligible, accounting for 0.2% of the total process global warming potential.^24^

**Figure 1:**
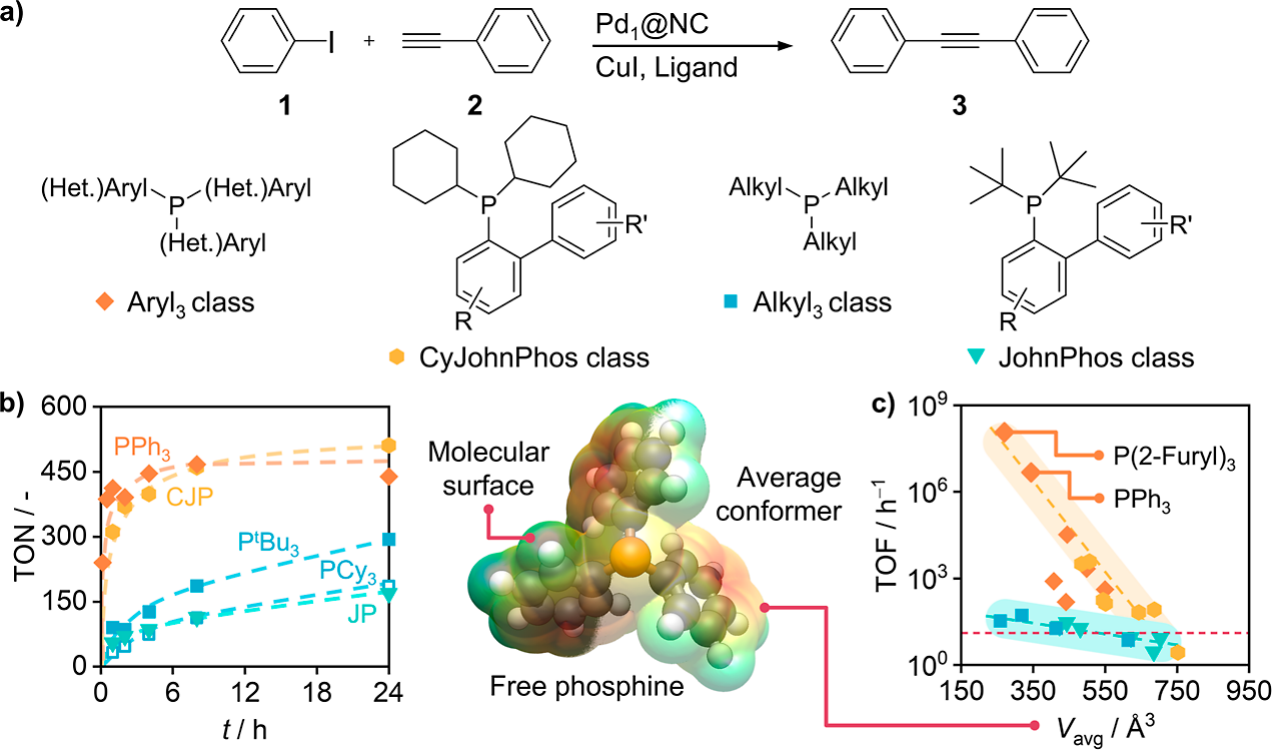
(a) Prototypical SH cross-coupling of iodobenzene (1) and ethynylbenzene (2), producing 1,2-diphenylethyne (3) and structural representations of 4 of the phosphine ligand classes that were used. (b) Time-resolved turnover number (TON) evolution for selected phosphines. (c) Turnover frequency (TOF) of Pd_1_@NC in the presence of phosphines exhibiting distinct average volumes of the free ligand. Adjacent to the plot is a 3D model of triphenylphosphine (PPh_3_, hydrogen: white, carbon: gray, phosphorus: orange) to visualize the average volume of the free ligands (*V*_avg_). Phosphine descriptors were openly accessible through the KRAKEN database. The symbols defined for each phosphine class in (a) apply to (b) and (c).

To gain deeper insights into the properties influencing the Pd-phosphine interaction, which remains elusive for SACs, we mapped the activity of Pd_1_@NC in the SH coupling in the presence of systematically chosen phosphines (Figures S1 and S2). An appropriate set to best represent their chemical space was proposed in 2022 by the founders of KRAKEN, a platform that provides open access to 190 computed descriptors (either for the free phosphine or as a ligand) for more than 1500 monodentate organophosphorus(III) compounds.^25,26^ The suggested list, known as the phosphine optimization screening set (PHOSS), was slightly modified for this work, primarily based on the commercial availability of the proposed compounds. To allow optimal comparability with literature-reported systems and transferability to more complex cross-coupling partner combinations, iodobenzene (1) and ethynylbenzene (2) were chosen for the SH coupling (Figure 1a). The Pd–Cu and Pd–P ratios as well as MeCN solvent and NEt_3_ base selection was based on previous results to yield optimal performance in the SH reaction over Pd_1_@NC.^24^ The comparably high amount of phosphine ligand (Pd/P ratio of 1:10) required is attributed to the dynamic nature of ligand interactions with the catalyst surface and copper halide. This adsorption/desorption of phosphine ligands with distinct sites on the palladium, copper, or nitrogen-doped carbon carrier impacts the effective ligand concentration near palladium centers, where only a single phosphine ligand can coordinate at any given point.

In the design of phosphine ligands for organometallic complexes classical approaches often introduce bulky functionalities to prevent overcoordination at the metal center, thereby enhancing performance. To evaluate whether this concept applies to SACs we monitored the reactivity of Pd_1_@NC in the presence of the modified PHOSS over time, determining the TOFs from the time to reach a TON of 100 (Table S1). These values were then compared to the respective average phosphine volume (*V*_avg_). Here, *V*_avg_ is defined as the volume that is enclosed by the molecular surface of the free phosphines’ Boltzmann average conformer.

The TON evolution (Figure 1b) shows no sign of an initiation phase for any of the SAC-phosphine systems. The TOF comparison reveals significant differences between trialkyl and JohnPhos (JP), and triaryl and CyJohnPhos (CJP) ligands (Figure 1c). In addition, a distinct behavior from that of molecular complexes is observed, with higher activity observed at smaller *V*_avg_ (Figure S3). This becomes particularly clear when comparing structurally related examples of the same class as those of JP or CJP. Adding more or bulkier substituents to the underlying base geometry of the phosphine leads to a decrease in activity.

This discloses a fundamental difference between organometallic complexes and SACs concerning the design principles of ligands. The reason is the support material, which occupies about half of the coordination sphere of each metal center and thus, only leaves enough space for a single phosphine molecule to coordinate. As such, the *V*_avg_ of the free phosphine determines its probability of reaching a suitable coordination distance to the metal center due to the steric interaction with the carrier. Using the *V*_avg_ as a descriptor for SH coupling activity, trifurylphosphine (P(2-furyl)_3_), an aryl_3_ affiliate, emerges as the optimal ligand, surpassing the previously employed PPh_3_. Meanwhile, classic descriptors like the cone angle or average buried volume of the ligand are unsuited to correlate the reactivity data. Plotting the TOF versus the minimum buried volume of the ligand in an organometallic complex (*V*_bur,min_) and the average energy of the P-substituent bond (*E*_σ*,P–R_) indicates the existence of activity thresholds (Figure S4). The presence of such thresholds has been used in recent studies concerning palladium-catalyzed cross-couplings using organometallic complexes to classify ligands as active or inactive.^27,28^ However, while these reports determined a necessary minimum *V*_bur,min_ of 57 Å^3^ to observe appreciable activity, here, we find that due to the presence of the solid support, a *V*_bur,min_ of 66 Å^3^ represents an upper limit for activity.

**Figure 2:**
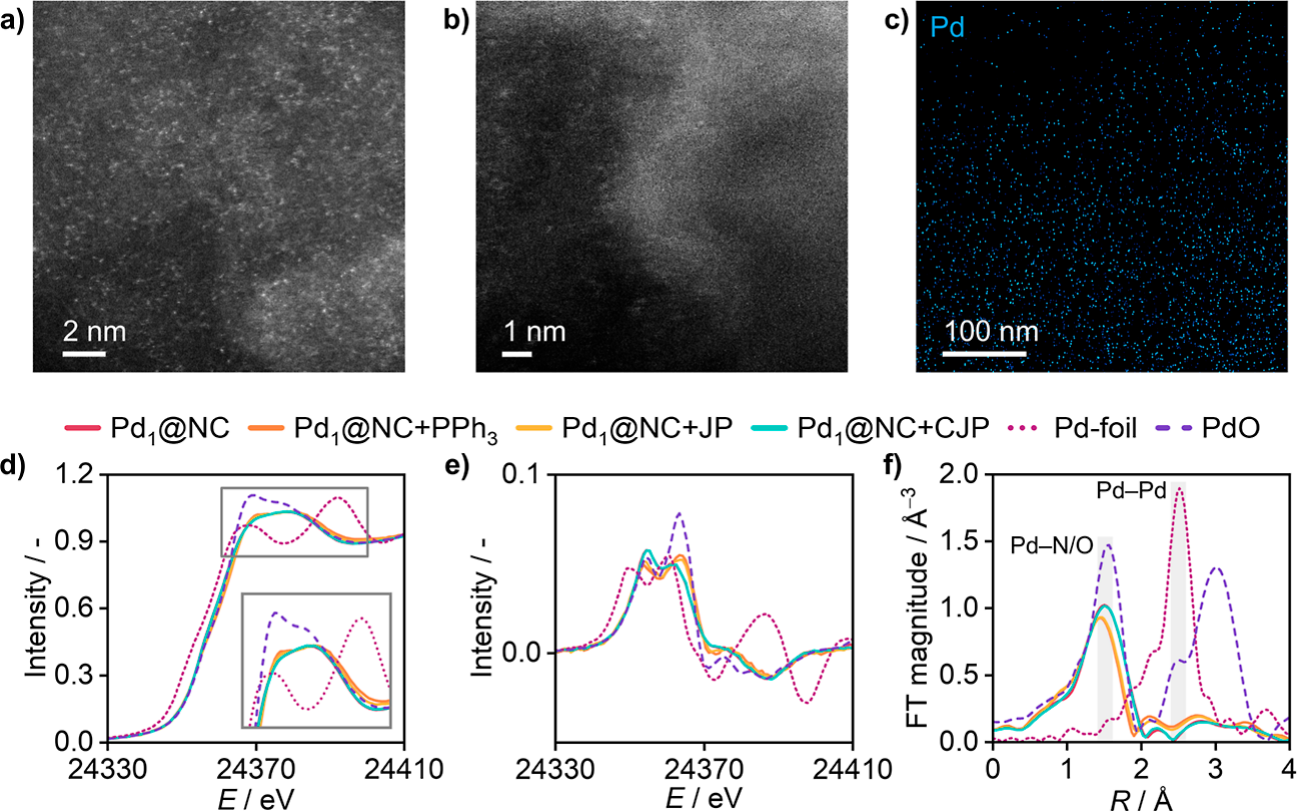
HAADF-STEM micrograph of the (a) as-prepared Pd_1_@NC and (b) washed-Pd_1_@NC. (c) EDX mapping of the washed-Pd_1_@NC. (d) Pd *K*-edge XANES spectra, (e) its first derivatives, and (f) FT-EXAFS (not corrected for phase-shift) corresponding to (a) of the Pd_1_@NC in MeCN in the absence of any phosphine and the presence of PPh_3_, JP and CJP. Palladium foil (magnitude ×0.5) and palladium(II)oxide were measured as references.

Analysis of the postreaction metal content in the Pd_1_@NC samples evidenced a minor palladium loss after application in the reaction. This may occur if weakly bound Pd species remain on the catalyst surface from its synthesis but withstand the removal through solvent rinsing. To verify that the results reflect the reactivity trends of the surface stabilized palladium centers and avoid any potential contribution of solubilized Pd species, the as-prepared Pd_1_@NC (Figure 2a) was subjected to a washing procedure before use (Figure 2b,c, Note S1). The reactivity trends observed in the evaluation of the washed-Pd_1_@NC after the second run matched those of the as-prepared Pd_1_@NC without any notable metal leaching, albeit at slower rates (Figure S5). Hot filtrations at the end of the reaction and catalyst recycling demonstrated its stability and verified that the data used in the comparison expressed the performance of surface-adsorbed active centers (Figures S6 and S7).

### Quasi-In Situ Analysis of the SAC-Phosphine Systems

The impact of the phosphine ligands on the electronic state of the palladium centers on the washed-Pd_1_@NC was characterized using XAS. Analysis of the X-ray absorption near edge structure (XANES) and its first derivative reveals the palladium being in an oxidation state close to Pd^II^ in PdO for Pd_1_@NC in the absence and the presence of phosphine (Figure 2d,e). This is an interesting result as the addition of phosphine is expected to lead to a reduction of the oxidation state of the palladium. When considering classical cross-coupling mechanisms for organometallic catalysts, it is broadly assumed, that the first step is a ligand-induced change of Pd^II^ into a Pd^0^, which then undergoes the oxidative addition.^29^ For carbon-supported SACs, recent reports indicate a similar process of electron donation from phosphorus toward the palladium to initiate the coupling reaction.^19^ Based on the Fourier-transformed extended X-ray absorption fine structure (FT-EXAFS) lacking a signal around 2.5 Å, it is safe to assume the absence of Pd–Pd bonding in any of the samples, supporting the single-atom configuration (Figure 2f). At the same time, the presence of Pd–P bonds cannot be verified either, due to the lack of a signal that would appear at 1.7 Å, as a shoulder of the Pd–N/O signal. The difficulty in verifying Pd–P interactions and changes in the electronic state of the palladium arises from the varying propensity of different palladium sites to adsorb phosphines. In the SAC, Pd atoms are stabilized by various coordination sites in the nitrogen-doped carbon carrier. However, the phosphine ligands can only coordinate to palladium centers with specific geometries (Table S2), vide infra, following section. Consequently, only a fraction of the metal centers interacts with the ligands. The structural diversity of the resulting palladium-phosphine configurations further complicates their detection. In addition, as the reference sample is measured in the presence of MeCN, interactions with the solvent could alter the electronic state of the palladium, making the differences between the absence and presence of ligands less pronounced. A similar inability to observe Pd–P interactions was noted in earlier studies on the Suzuki–Miyaura cross-coupling despite evidence of electronic state changes of palladium.^19^ This problem can be potentially targeted by aiming for higher metal contents in the samples as well as utilizing fluorescence mode for XAS data acquisition, providing a lower detection limit and greater surface sensitivity. Still, a systematic investigation of the activity contributions from all components of the catalytic system in a previous study strongly suggests that Pd–P interactions are responsible for the reactivity trends observed across the different ligands.^24^ In the absence of ligands, only negligible reactivity toward the desired SH product was seen. Significant activity was only observed when both Pd_1_@NC and phosphine were present. CuI exhibited no appreciable reactivity, either alone or in combination with PPh_3_, NC, or PPh_3_ and NC.

### Reactivity Descriptor Identification

To understand the distinct trends observed with different phosphine ligands and rationalize the key descriptors that govern the performance of the SAC-phosphine ensemble, we used a DFT-based approach to shed light on the codependent interactions. Accordingly, 5 phosphines were chosen to analyze their coordination and subsequent effects on the activation of the palladium center in Pd_1_@NC. These include PPh_3_ as a benchmark based on its broad use in literature protocols, JP and CJP for their distinct activity, and P^*t*^Bu_3_ and PCy_3_ for their structural relation to JP and CJP (Figures S8 and S9). The square planar tetrapyridinic (4 × N6, Pyri_4_) and dipyridinic-dipyrrolic (2 × N6 + 2 × N5, Pyr_2+2_), as well as trigonal planar tripyrrolic (3 × N5, Pyrr_3_) cavities on the NC surface were chosen as representative metal coordination sites for the NC support (Figure S10).^30,31^ The choice of these cavities is in line with literature, that used a combination of X-ray spectroscopy and computational simulations to determine the type, abundance, and arrangement of nitrogen moieties in a nitrogen-doped carbon.^32^

**Figure 3:**
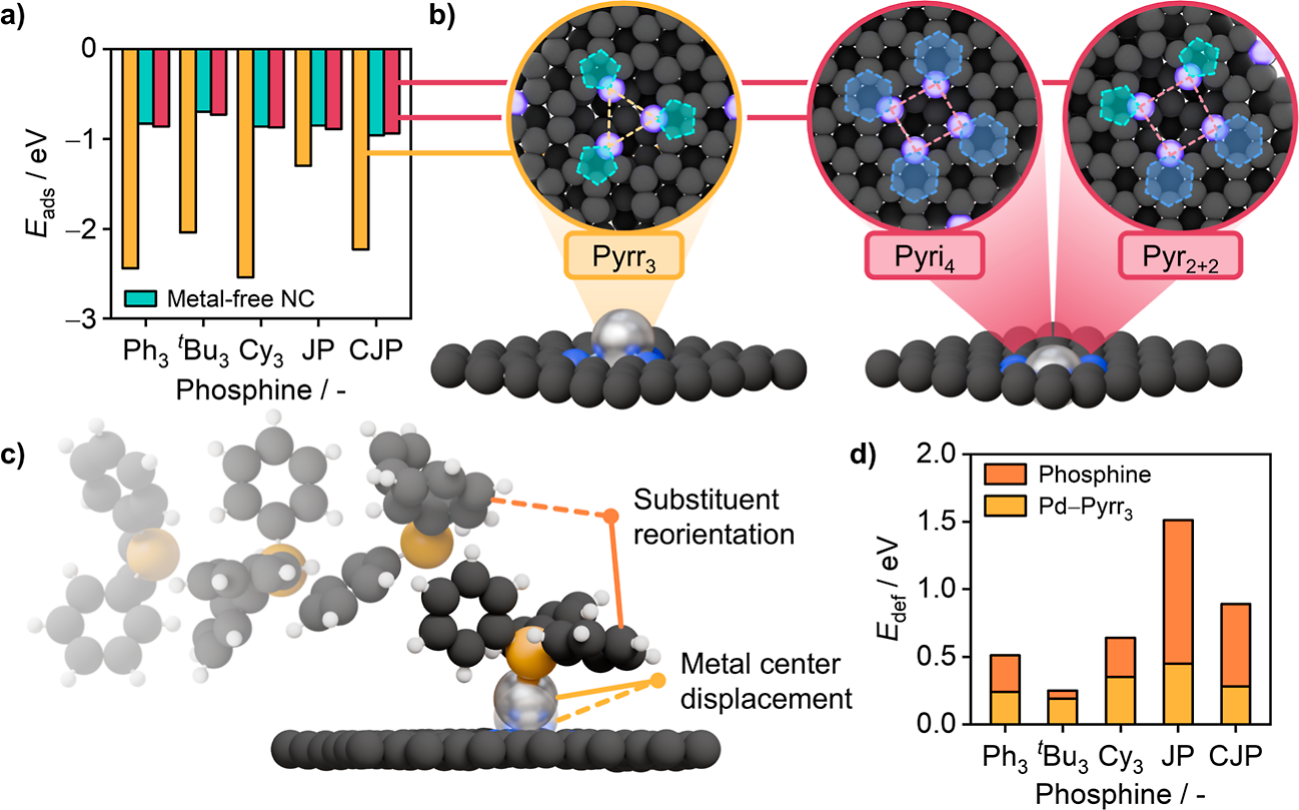
(a) Adsorption energies (*E*_ads_) of PPh_3_ (Ph_3_), P^*t*^Bu_3_ (^*t*^Bu_3_), PCy_3_ (Cy_3_), JP and CJP on the metal-free NC carrier (green) and (b) Pyrr_3_ (yellow) or Pyri_4_ and Pyr_2+2_ (red, representative for both cavities) stabilized palladium centers. (c) 3D visualization of PPh_3_ coordinating to the palladium center while reorienting its substituents (phosphine deformation) as well as displacing the palladium (metal site deformation; hydrogen: white, carbon: dark gray, phosphorus: orange). (d) Deformation energies (*E*_def_) of the metal site and the phosphines considered in (a) during coordination to a Pyrr_3_-stabilized palladium atom.

Geometry inspection and analysis of the adsorption energy (*E*_ads_) obtained from the simulation of the phosphines adsorbed on the SAC revealed a preference for a Pd–P interaction only for the center stabilized in the Pyrr_3_ cavity (Figure 3a), for which the *E*_ads_ values range from −1.30 up to −2.54 eV. The tetrahedral Pd–N geometry in this cavity leads to a more accessible out-of-plane coordinated palladium center, enabling interaction with the phosphines (Figure 3b). The adsorbed configuration for PCy_3_ exhibits the highest stabilization because of the direct interaction of the cyclohexyl hydrogen atoms with the metal center. CJP and PPh_3_ experience enhanced stabilization, likely due to dispersion contributions between hydrogen atoms of phosphine and the graphitic layer, and π–π stacking contributions.

For the Pyri_4_ and Pyr_2+2_ cavities, geometry inspection shows no signs of a Pd–P coordination (Figures S11–S13). This is further corroborated by the *E*_ads_ values for these sites, ranging from −0.72 to −0.96 eV which is comparable to those calculated for phosphine adsorption on the metal-free NC surface (Table S2). We attribute the inability of the phosphines to coordinate to the palladium atoms in the square planar cavities to the in-plane overstabilization of the metal, originating from the higher number of nitrogen atoms and greater cavity size. The selective coordination of the phosphines would also explain the difficulties of detecting a change in the electronic state of the metal in the quasi-in situ XAS measurements, as only a part of the metal atoms is able to interact.

To understand the role of the ligand in the SAC performance enhancement, we characterized the SAC–phosphine structure through electronic and geometric terms. Starting from the electronic state of the phosphorus in the SAC–phosphine configurations, a charge analysis was performed for the metal and phosphorus atoms. Results showed variations in Bader charges (*q*_Bader_) for the phosphorus atoms, ranging from 0.82 for P^*t*^Bu_3_ to 1.60 for PPh_3_ (Table S3). These suggest that the catalytic activity is affected by the distinct capabilities of the phosphorus to provide electron density for the metal center. Yet, by analyzing *q*_Bader_ of the metal center we found a reduction from 0.72 to ∼0.50 across all examples, due to the modulation of the metal’s electronic state by the extended aromatic network of the carrier.^33^ This describes a substantial difference to molecular catalysts in which the choice of the phosphine substituents is also aimed at tailoring the electronic state of the palladium, usually to enhance the rate of oxidative addition.

We then shifted our focus toward the steric properties linked to the SAC–phosphine geometries. Interestingly, the simulations showed an increase in the metal center-carrier distance (*d*_Pd-carrier_) by about 30% through elongation of the Pd–N bonds, while maintaining the coordination to the anchoring nitrogen atoms. This was found in all cases during the phosphine adsorption at the palladium, regardless of the phosphine structure (Table S4). The increase in *d*_Pd-carrier_ facilitates access of cross-coupling reagents to the active site, making it another key mechanism in the activation of the palladium besides the charge reduction.

To quantify the energetic cost of the activation, the deformation energy (*E*_def,i_ = *E*_i_(total geom.) – *E*_i_(free) for i = Pd, P) was determined (Figure 3c). The first term consists of the deformation of the metal site (*E*_def,Pd_), which accounts for the required energy to displace the metal atom during the Pd–P coordination. Despite the similarities in the *d*_Pd-carrier_ increase, *E*_def,Pd_ features variations of more than 100%. This is a result of an energetically unfavorable asymmetric elongation of the three Pd–N bonds (horizontal displacement) that the palladium atom experiences.

The second term considers the energy requirement of reorienting the phosphine’s substituents (*E*_def,P_) when approaching the catalyst. A comparison of the *E*_def,P_ shows that the energy penalty is generally higher the more complex the ligands are, ranging from 0.06 eV for P^*t*^Bu_3_ to 1.06 eV for JP (Table S5). The disparity in *E*_def,P_ between JP (1.06 eV) and CJP (0.60 eV) phosphines elucidates the difference in catalytic activities as the ^*t*^Bu bulk impedes rotation of the biphenyl group, energetically penalizing the adsorption of JP to the metal center. However, it does not explain the poor performance of the systems utilizing trialkyl ligands, as these exhibit *E*_def_ values that are even lower than that of the CJP.

**Figure 4:**
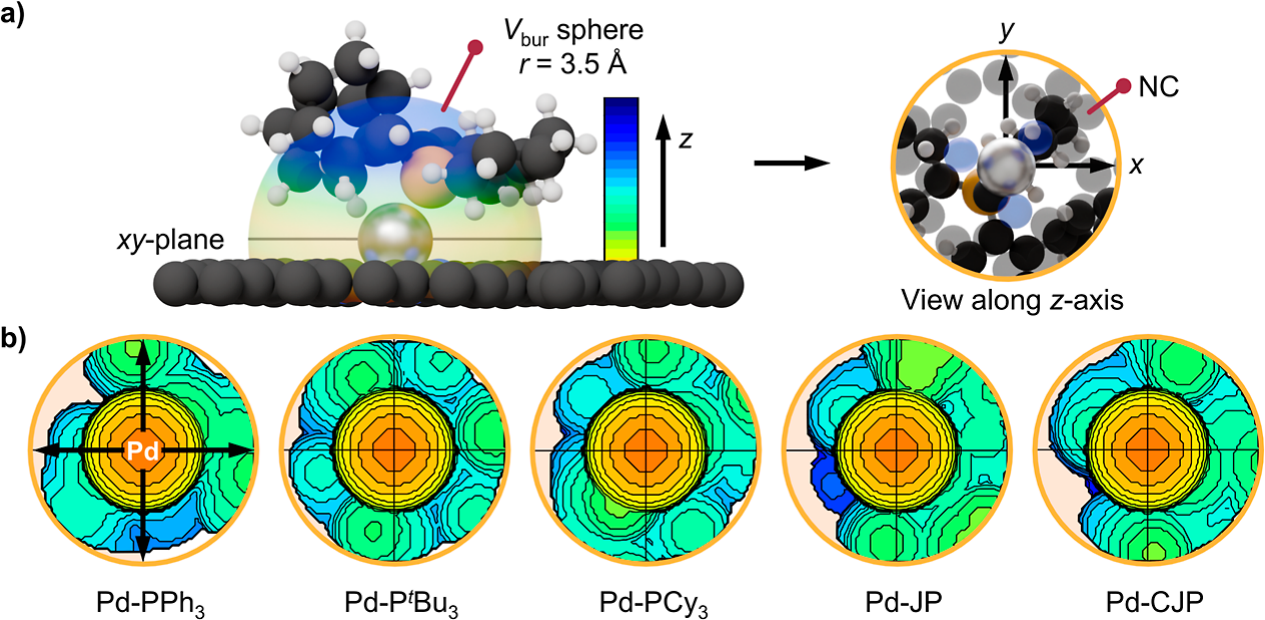
(a) 3D representation of the palladium-centered buried volume sphere (*V*_bur_ sphere) used to generate the topographic heatmaps. The *xy*-plane (gray line) is parallel to the support surface plane and the *z*-axis is perpendicular. (b) The resulting topographic heatmaps of the Pd-ligand systems at the Pyrr_3_ metal coordination site, using the color scale shown in the legend of (a) to indicate the location of atoms within the buried volume sphere along the *z*-axis. Phosphine ligand nomenclature as in Figure 3.

**Figure 5:**
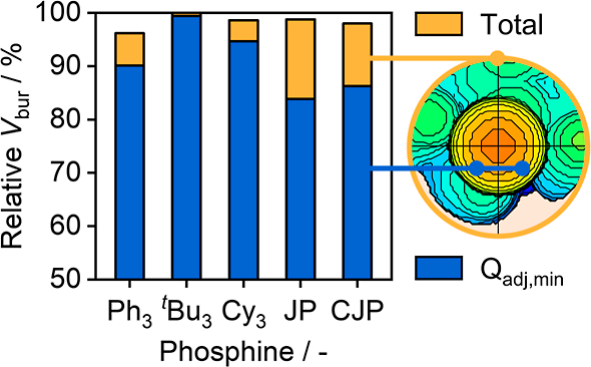
Comparison of the total (yellow) buried volume (*V*_bur_) and adjacent quadrant pairs of minimal *V*_bur_ (*Q*_adj,min_, blue) in the Pd_Pyrr3_–phosphine hemispheres for the SAC-ligand systems. The total *V*_bur_ of P^*t*^Bu_3_ is set as 100%, as it exhibits the greatest value.

In the Pd_Pyrr3_–phosphine geometry following the ligand-induced palladium activation, the hemisphere of the palladium coordination sphere above the carrier plane is occupied by the phosphine, making the accessibility of the cross-coupling reagents to the palladium atom key to describing its reactivity. Therefore, we determined the *V*_bur_ at the active center for the different SAC–phosphine systems (Tables S6–S8). With the help of SambVca,^34^ a software tool developed for organometallic complexes, the volume occupation was calculated within spheres of 3.5, 4.5, or 6.5 Å radius, considering the host as a metal ligand, and displayed as two-dimensional, quadrant-separated topographic heatmaps (Figures 4 and S14, S15). The evaluation of the total *V*_bur_ in the Pd_Pyrr3_–phosphine hemisphere (average *V*_bur_ of the four hemisphere quadrants) disclosed a similar spatial demand at the center for all examples (Figure 5). However, by analyzing the quadrants with the lowest *V*_bur_ we found that the JP and CJP exhibit contiguous areas (*Q*_adj,min_) in which the steric shielding of the active site is almost 20% lower than that of the alkyl_3_ analogs. This difference arises from the interaction of the flat and rigid aromatic biphenyl group with the NC, which forces the biphenyl moiety away from the carrier plane, exposing the metal atom. Meanwhile, the alkyl_3_ phosphine substituents present a symmetric umbrella-like coverage that hampers access to the active site.

**Figure 6:**
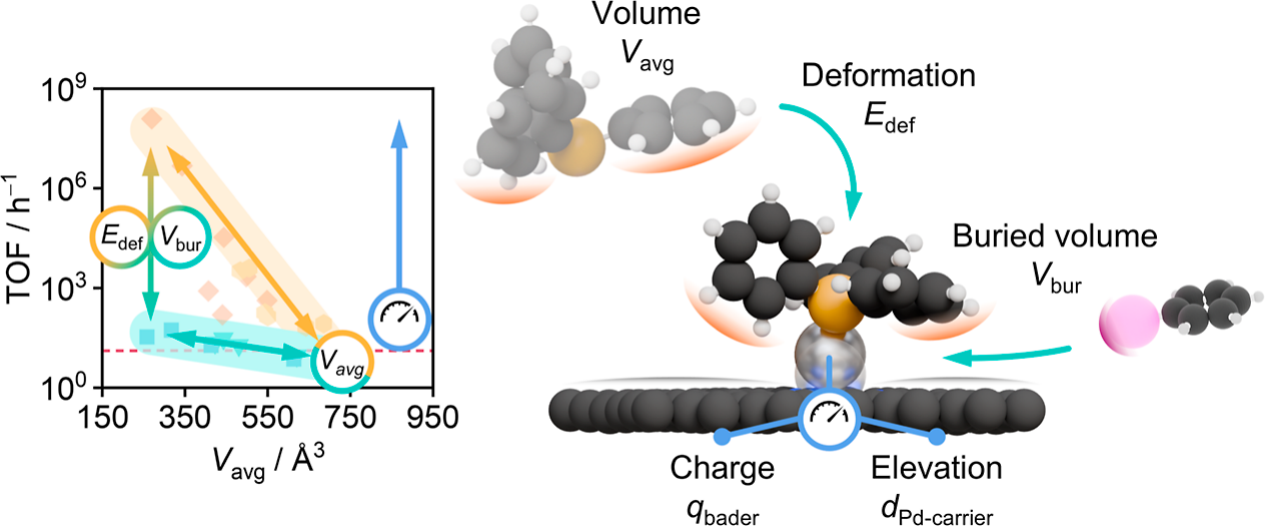
Interplay of the distinct properties of the SAC-phosphine system that have been identified to govern the reactivity (Figure 1).

Based on these results, we can attribute the performance trends that were observed in the reactivity tests to three main properties of the ligand (Figure 6). The first is the *V*_avg_, which, based on the repulsive interaction with the carrier, affects the ability of the ligand to get in proximity to the metal. Afterward, it is the *E*_def_, quantifying the complexity of rearranging the phosphine substituents and active site during coordination to the metal atom. Finally, it is the *V*_bur_ in the coordination environment of the palladium, governing the accessibility of the coupling materials to the metal atom. All of these are strongly affected by the solid support which imposes substituent reorientation on the phosphine and restricts the palladium coordination sphere. The differences in the electron donation capabilities of the phosphines were found to be negligible. While it is important to reduce the metal atom to enable oxidative addition, possible variations in the electronic state of the metal are compensated for by the carrier’s extended aromatic network. Simulations show that *d*_Pd–P_ and *d*_Pd–N_ increase significantly (Table S9) during the adsorption of starting material at metal centers activated by the well-performing ligands PPh_3_ and CJP (Figure S16). This adaptive bonding behavior, driven by the electronic structure of the carbon carrier, reduces the coordinative strain and allows reorientation of the aryl halide at the metal center. Once the palladium inserts into the I–C bond the coupling is initiated. After desorption of the product during the reductive elimination, the palladium center returns to its stable, support-bound state.

## Conclusions

This work establishes the key role of ligands in activating palladium centers in SACs for cross-coupling reactions. A comprehensive evaluation of a SAC in combination with a diverse set of phosphine ligands in the SH reaction underscores the strong reactivity enhancement that can be achieved. Differently from organometallic complexes, we identified a clear correlation between activity and decreasing average volume of the free ligand conformer, pinpointing P(2-furyl)_3_ as a superior alternative to the commonly used PPh_3_. Notably, CyJohnPhos and aryl_3_ ligands exhibited a more pronounced promotional effect than those of the alkyl_3_ and JohnPhos families. Quasi-in situ XAS studies demonstrated that, unlike organometallic catalysts, the choice of ligand does not significantly modify the electronic properties of palladium centers in the Pd_1_@NC SAC, due to the modulation through the extended aromatic network of the functionalized carbon support. DFT simulations suggest that the primary activating mechanism of ligands stems from the improved accessibility of metal centers, facilitated by a subtle displacement from their metal coordination sites. Furthermore, our analysis of the geometric properties of SAC-phosphine systems revealed that the promotional effect is driven by both the deformation energy of the ligand between the free and adsorbed states, favored by lower values, and enhanced accessibility of metal centers with fewer phosphine atoms residing in their buried volume shell. The findings emphasize the important role of ligands and open new avenues for tailoring the 3D atomic environments of SACs to optimize their performance.

## Methods

Nitric acid (>65 wt %, puriss.) and dicyandiamide (99%) were purchased from Sigma-Aldrich, activated carbon (AC, Norit Rox 0.8) from Cabot Corporation, and Pd(NO_3_)_2_·2H_2_O (41 wt % Pd) from abcr. The reagents for the cross-coupling reactions were purchased from Chemie Brunschwig AG. All chemicals were used without further purification.

### Preparation of Pd_1_@NC

For the nitrogen incorporation of the carrier, AC was sieved (sieve fraction <0.2 mm) and refluxed in nitric acid (4 M, 20 cm^3^ g_AC_^–1^) at 353 K for 16 h. The mixture was poured into DI water (273 K, 20 cm^3^ g_AC_^–1^), filtered, washed copiously with DI water (0.2 dm^3^ g_AC_^–1^), and dried overnight (338 K). The acid-activated carbon was added to a solution of dicyandiamide (3 g g_AC_^–1^) in acetone (0.3 dm^3^ g_AC_^–1^), which was subsequently evaporated at 353 K under constant stirring. Finally, the dried solid was gently crushed, transferred to ceramic boats, and carbonized in flowing nitrogen (723 K, 3 h hold, then 923 K, all ramps 5 K min^–1^) to obtain nitrogen-doped carbon (NC, 2.4 g g_AC_^–1^) as a black powder. Pd(NO_3_)_2_·2H_2_O (5 mg g_NC_^–1^) and DI water (4 cm^3^ g_NC_^–1^) were added to a sonicated (30 min) suspension of as-prepared NC in DI water (12 cm^3^ g_NC_^–1^) and stirred overnight. After filtration, the solids were washed with DI water (120 cm^3^ g_NC_^–1^) and dried at 383 K. Finally, the solid was annealed in a static nitrogen atmosphere (573 K, 5 h, 5 K min^–1^ ramp) to obtain the Pd_1_@NC (∼1 g g_NC_^–1^) as a black solid. The protocol’s applicability was verified up to a Pd_1_@NC production scale of 50 g.^24^

### Catalyst Characterization

The metal content was analyzed by inductively coupled plasma optical emission spectroscopy using a Horiba Ultra 2 instrument (photomultiplier tube detector). Sample aliquots (15 mg) were subjected to a microwave digestion treatment (473 K, 20 min, 48 bar) using concentrated nitric acid (>65 wt %, 3 cm^3^) to dissolve the matrix. The obtained solutions were diluted with Milli-Q water and solids were removed through polytetrafluoroethylene (PTFE) syringe filters (0.25 μm pore size). For scanning transmission electron microscopy (STEM), the samples were dusted onto carbon-film copper and nickel grids (300 mesh). High-angle annular dark field scanning transmission electron microscopy (HAADF-STEM) and energy dispersive X-ray spectroscopy (EDX) measurements were performed on a Talos F200X instrument operated at 200 kV and equipped with an FEI SuperX detector. High magnification micrographs were acquired on a JEOL GrandARM operated at 300 kV. EDX elemental maps were averaged over 5 frames (1024 × 1024 pixel, 15 ms pixel dwell time) in the spectral range up to 20 keV, and postprocessed (background subtraction and Gaussian blur). XAS was conducted at the BM31 (SNBL) beamline of the European Synchrotron Radiation Facility (ESRF) in Grenoble, France. The washed-Pd_1_@NC was added to a cylindrical polypropylene (PP) vessel alone (reference) or in combination with the phosphines (Pd/P, 1:10) PPh_3_, JP, or CJP, and treated with MeCN. To avoid oxidation of the phosphines, the samples were prepared in a glovebox using molecular nitrogen as an inert gas. With the low palladium content (0.22 wt % Pd) of the catalyst, the vessel was chosen with a wall thickness of 1 mm and a beam path length through the sample of 1 cm to acquire data of suitable quality, and the measurement was performed in transmission mode aiming for a better signal-to-noise ratio. The X-ray beam was monochromatized using an air-bearing liquid nitrogen double-crystal monochromator (Si[111]) and collimated to a size of 3 mm × 200 μm (horizontal × vertical). Data were acquired at the palladium *K*-edge (*E*_0_ = 24.35 keV) in transmission (200 mA synchro functioning) mode, using Ar/N_2_-filled ionization chambers. The samples were placed between the first and the second ionization chamber. For the absolute energy calibration, palladium foil was measured simultaneously between the second and third ionization chambers. The resulting spectra were energy calibrated, background corrected, and normalized using the Athena program from the Demeter software suite.^35^ To prepare the samples, the catalyst and phosphine were mixed in acetonitrile and added to a snap-cap vessel (1 cm diameter, polypropylene) in a glovebox, before sealing the opening using an epoxy resin.

### Catalyst Evaluation

Unless otherwise stated, the SH coupling reaction was performed following a standard procedure: a degassed solution (2.53 g) consisting of iodobenzene (1, 12.4 wt %, 1.0 equiv, equiv), ethynylbenzene (2, 9.20 wt %, 1.4 equiv), 1,3,5-trimethylbenzene (2.9 wt %, internal standard) and acetonitrile (MeCN, 75.5 wt %) was added to a screw cap glass vial (8 cm^3^) containing the palladium catalyst (0.53 wt % Pd, 60 mg 0.2 mol %) and phosphine (2.0 mol %), followed by the addition of a freshly prepared and degassed solution (2.34 g) of copper(I) iodide (CuI, 0.47 wt %, 4.0 mol %), triethylamine (NEt_3_, 19.4 wt %, 3.0 equiv), and MeCN (80.1 wt %). The resulting suspension was vigorously stirred for 24 h at 353 K under a protective atmosphere (Ar), cooled to room temperature afterward and the SAC separated from the reaction mixture by filtration. Hot filtration was performed by transferring the hot reaction mixture into a syringe and separating the catalyst immediately by filtering the mixture through a CHROMAFIL Xtra PTFE (20/25, 0.20 μm pore size) syringe filter. The solution was transferred into a fresh vessel and continued to stir afterward without further treatment. The reaction solution was analyzed by gas chromatography flame ionization detection (GC-FID). The catalyst turnover number (TON) was calculated by dividing the number of product molecules present in the mixture by the number of Pd atoms that were initially added to the reaction. The turnover frequency (TOF) was calculated by dividing a TON of 100 by the time (*t*_100_) necessary for the system to reach it (TOF = TON × *t*^–1^ = 100 × *t*_100_^–1^). The specific *t*_100_ for each SAC-phosphine combination was estimated by monitoring the evolution of 1,2-diphenylethyne (3) yield in the SH coupling (Figures S1 and S2) and interpolating these data.

To simplify the workflow for condition screenings, stock solutions consisting of iodobenzene (1), ethynylbenzene (2), internal standard, and MeCN as well as NEt_3_ and MeCN were prepared. If properly degassed by at least three freeze–pump–thaw cycles and kept under an inert atmosphere afterward, the stock solutions could be stored for multiple weeks without any change in composition. This was monitored by GC-FID and a reference (*t* = 0) sample taken before the use of the respective stock solution as a comparison to the postreaction analysis. GC-FID was performed on a Thermo TRACE 1300 chromatograph equipped with a flame ionization detector, and a ZB-5 column (5%-phenyl-95%-dimethylpolysiloxane, 30 m length, 0.25 mm inner diameter, 0.25 mm film thickness) using helium as carrier gas. An overview of the employed phosphorus compounds as well as the results of the recycling experiments and time resolution of the reaction progress are reported in the Supporting Information (Table S1, Figures S1–S7, Scheme S1).

### Computational Details

DFT simulations were performed using the Vienna ab initio simulation package (VASP, version 5.4.4) to gain further insight into the metal-carrier complex when interacting with different phosphine families.^36,37^ The generalized gradient approximation of the Perdew–Burke–Ernzerhof (GGA PBE) functional was used to obtain the exchange–correlation energies including dispersion via D3.^38,39^ The projector augmented wave method (PAW) was used to describe inner electrons and plane waves were used for valence electrons with a cutoff energy of 450 eV.^40^ Following our works on similar systems to account for the range of possible environments for the Pd_1_@NC, three different cavities were considered: square planar tetrapyridinic (4 × N6, Pyri_4_), dipyridinic-dipyrrolic (2 × N6 + 2 × N5, Pyr_2+2_) and trigonal planar tripyrrolic (3 × N5, Pyrr_3_).^30^ The NC, Pd_1_@NC, and Phosphine–Pd_1_@NC systems were modeled as a monolayer of graphitic carbon, using the same box and vacuum parameters, and *k*-points as the single-atom catalysts. The SACs were simulated by constructing a monolayer slab in a 14.8 × 14.8 × 19 Å^3^ box with a Γ-centered mesh of 3 × 3 × 1 *k*-points. Conformer generation for gas-phase phosphines was performed with CREST at the GFN-xTB level, and the lowest 3 energy conformers were optimized with DFT using Gaussian16 at the B3LYP theory level with the 6-31G(d,p) basis set for all atoms and including Grimme’s D3 dispersion.^41−46^ All the optimized molecules were characterized as minima of their corresponding potential energy surfaces by analysis of the eigenvalues of the diagonalized Hessian matrices. These conformers were then recomputed with VASP at PBE + D3 in a box of 15 × 15.5 × 16 Å^3^ using a single *k*-point.

The analysis of the buried volume (*V*_bur_) was performed for the Pd-phosphine and Pd-carrier systems using the SambVca 2.1 software, considering a sphere centered on the palladium atom with a radius of 3.5, 4.5, or 6.5 Å and defining the *z*-axis perpendicular and the *x*- and *y*-axis parallel to the support.^34^ Default bond radii scaling by 1.17 and mesh spacing for numerical integration of 0.1 Å have been employed. Hydrogen atoms have been considered when computing volumes. The %*V*_bur,total_ was calculated by dividing the determined *V*_bur_ by the hemisphere volume (*V*_hemisphere_ = *V*_sphere,*V*bur_ × 0.5) of the *V*_bur_ sphere and multiplying it by 100 (%*V*_bur,total_ = *V*_bur_ × *V*_hemisphere_^–1^ × 100). The %*V*_bur,Qx_ was calculated by dividing the *V*_bur_ of the phosphine within one of the hemisphere quadrants (Q_*x*_, *x* = 1, 2, 3, or 4) by the quadrant volume (*V*_quadrant_ = *V*_sphere,*V*bur_ × 0.125) and multiplying it by 100 (%*V*_bur,Qx_ = *V*_bur_ × *V*_quadrant_^–1^ × 100).

The values for all parameters investigated, 3D representations of cavities and adsorbed geometries as well as the generated topographic heatmaps can be found in the Supporting Information (Tables S2–S8, Figures S8–S15).

## Data Availability

The experimental and computational data sets presented in this study are openly available on the Zenodo and ioChem-BD databases, respectively.^47,48^

## Supporting Information Available

Additional experimental procedure for the catalyst washing, results of the SAC evaluation in the SH cross-coupling and DFT simulations, and visualizations of the SAC–phosphine configurations (PDF)

## References

[ref1] Chen F.; Jiang X.; Zhang L.; Lang R.; Qiao B. Single-Atom Catalysis: Bridging the Homo- and Heterogeneous Catalysis. Chin. J. Catal. 2018, 39 (5), 893–898. 10.1016/S1872-2067(18)63047-5.

[ref2] Arango-Daza J. C.; Rivero-Crespo M. A. Multi-Catalytic Metal-Based Homogeneous-Heterogeneous Systems in Organic Chemistry. Chem.—Eur. J. 2024, 202400443 10.1002/chem.202400443. 38958991

[ref3] Christmann U.; Vilar R. Monoligated Palladium Species as Catalysts in Cross-Coupling Reactions. Angew. Chem., Int. Ed. 2005, 44 (3), 366–374. 10.1002/anie.200461189. 15624192

[ref4] Wolfe J. P.; Buchwald S. L. A Highly Active Catalyst for the Room-Temperature Amination and Suzuki Coupling of Aryl Chlorides. Angew. Chem., Int. Ed. 1999, 38 (16), 2413–2416. 10.1002/(SICI)1521-3773(19990816)38:16<2413::AID-ANIE2413>3.0.CO;2-H. 10458806

[ref5] Littke A. F.; Dai C.; Fu G. C. Versatile Catalysts for the Suzuki Cross-Coupling of Arylboronic Acids with Aryl and Vinyl Halides and Triflates under Mild Conditions. J. Am. Chem. Soc. 2000, 122 (17), 4020–4028. 10.1021/ja0002058.

[ref6] Roughley S. D.; Jordan A. M. The Medicinal Chemist’s Toolbox: An Analysis of Reactions Used in the Pursuit of Drug Candidates. J. Med. Chem. 2011, 54 (10), 3451–3479. 10.1021/jm200187y. 21504168

[ref7] Van Velthoven N.; Wang Y.; Van Hees H.; Henrion M.; Bugaev A. L.; Gracy G.; Amro K.; Soldatov A. V.; Alauzun J. G.; Mutin P. H.; De Vos D. E. Heterogeneous Single-Site Catalysts for C-H Activation Reactions: Pd(II)-Loaded S,O-Functionalized Metal Oxide-Bisphosphonates. ACS Appl. Mater. Interfaces 2020, 12 (42), 47457–47466. 10.1021/acsami.0c12325. 32970411

[ref8] Sharma S.; Gallou F.; Handa S. Towards a Sustainable Tomorrow: Advancing Green Practices in Organic Chemistry. Green Chem. 2024, 26 (11), 6289–6317. 10.1039/D4GC01826E.

[ref9] Bryan M. C.; Dunn P. J.; Entwistle D.; Gallou F.; Koenig S. G.; Hayler J. D.; Hickey M. R.; Hughes S.; Kopach M. E.; Moine G.; Richardson P.; Roschangar F.; Steven A.; Weiberth F. J. Key Green Chemistry Research Areas from a Pharmaceutical Manufacturers’ Perspective Revisited. Green Chem. 2018, 20 (22), 5082–5103. 10.1039/C8GC01276H.

[ref10] Faust Akl D.; Poier D.; D’Angelo S. C.; Araújo T. P.; Tulus V.; Safonova O. V.; Mitchell S.; Marti R.; Guillén-Gosálbez G.; Pérez-Ramírez J. Assessing the Environmental Benefit of Palladium-Based Single-Atom Heterogeneous Catalysts for Sonogashira Coupling. Green Chem. 2022, 24 (18), 6879–6888. 10.1039/D2GC01853E. 36276229 PMC9487187

[ref11] Zhang Y.; Ye S.; Gao M.; Li Y.; Huang X.; Song J.; Cai H.; Zhang Q.; Zhang J. N-Doped Graphene Supported Cu Single Atoms: Highly Efficient Recyclable Catalyst for Enhanced C–N Coupling Reactions. ACS Nano 2022, 16 (1), 1142–1149. 10.1021/acsnano.1c08898. 36350100

[ref12] Singh B.; Gawande M. B.; Kute A. D.; Varma R. S.; Fornasiero P.; McNeice P.; Jagadeesh R. V.; Beller M.; Zbořil R. Single-Atom (Iron-Based) Catalysts: Synthesis and Applications. Chem. Rev. 2021, 121 (21), 13620–13697. 10.1021/acs.chemrev.1c00158. 34644065

[ref13] Barlocco I.; Di Liberto G.; Pacchioni G. Hydrogen and Oxygen Evolution Reactions on Single Atom Catalysts Stabilized by a Covalent Organic Framework. Energy Adv. 2023, 2 (7), 1022–1029. 10.1039/D3YA00162H.

[ref14] Wang A.; Li J.; Zhang T. Heterogeneous Single-Atom Catalysis. Nat. Rev. Chem 2018, 2 (6), 65–81. 10.1038/s41570-018-0010-1.

[ref15] Büchele S.; Chen Z.; Mitchell S.; Hauert R.; Krumeich F.; Pérez-Ramírez J. Tailoring Nitrogen-Doped Carbons as Hosts for Single-Atom Catalysts. ChemCatChem 2019, 11 (12), 2812–2820. 10.1002/cctc.201900547.

[ref16] Chen Z.; Vorobyeva E.; Mitchell S.; Fako E.; Ortuño M. A.; López N.; Collins S. M.; Midgley P. A.; Richard S.; Vilé G.; Pérez-Ramírez J. A Heterogeneous Single-Atom Palladium Catalyst Surpassing Homogeneous Systems for Suzuki Coupling. Nat. Nanotechnol. 2018, 13 (8), 702–707. 10.1038/s41565-018-0167-2. 29941887

[ref17] Kim S.; Bok J.; Lee B.-H.; Choi H.; Seo Y.; Kim J.; Kim J.; Ko W.; Lee K.-S.; Cho S.-P.; Hyeon T.; Yoo D. Orthogonal Dual Photocatalysis of Single Atoms on Carbon Nitrides for One-Pot Relay Organic Transformation. ACS Nano 2023, 17 (21), 21470–21479. 10.1021/acsnano.3c06314. 37847158

[ref18] Zhao Q.; Zhao X.; Liu Z.; Ge Y.; Ruan J.; Cai H.; Zhang S.; Ye C.; Xiong Y.; Chen W.; Meng G.; Liu Z.; Zhang J. Constructing Pd and Cu Crowding Single Atoms by Protein Confinement to Promote Sonogashira Reaction. Adv. Mater. 2024, 36 (36), 2402971 10.1002/adma.202402971. 39011789

[ref19] Moragues T.; Giannakakis G.; Ruiz-Ferrando A.; Borca C. N.; Huthwelker T.; Bugaev A.; de Mello A. J.; Pérez-Ramírez J.; Mitchell S. Droplet-Based Microfluidics Reveals Insights into Cross-Coupling Mechanisms over Single-Atom Heterogeneous Catalysts. Angew. Chem., Int. Ed. 2024, 63 (20), e202401056 10.1002/anie.202401056. 38472115

[ref20] Tolman C. A. Electron Donor-Acceptor Properties of Phosphorus Ligands. Substituent Additivity. J. Am. Chem. Soc. 1970, 92 (10), 2953–2956. 10.1021/ja00713a006.

[ref21] Tolman C. A. Steric Effects of Phosphorus Ligands in Organometallic Chemistry and Homogeneous Catalysis. Chem. Rev. 1977, 77 (3), 313–348. 10.1021/cr60307a002.

[ref22] Fleckenstein C. A.; Plenio H. Sterically Demanding Trialkylphosphines for Palladium-Catalyzed Cross Coupling Reactions—Alternatives to PtBu_3_. Chem. Soc. Rev. 2010, 39 (2), 694–711. 10.1039/B903646F. 20111788

[ref23] Jeong H.; Shin S.; Lee H. Heterogeneous Atomic Catalysts Overcoming the Limitations of Single-Atom Catalysts. ACS Nano 2020, 14 (11), 14355–14374. 10.1021/acsnano.0c06610. 33140947

[ref24] Poier D.; Akl D. F.; Lucas E.; Machado A. R.; Giannakakis G.; Mitchell S.; Guillén-Gosálbez G.; Marti R.; Pérez-Ramírez J. Reaction Environment Design for Multigram Synthesis via Sonogashira Coupling over Heterogeneous Palladium Single-Atom Catalysts. ACS Sustain. Chem. Eng. 2023, 11 (48), 16935–16945. 10.1021/acssuschemeng.3c04183. 38076617 PMC10698743

[ref25] Gensch T.; dos Passos Gomes G.; Friederich P.; Peters E.; Gaudin T.; Pollice R.; Jorner K.; Nigam A.; Lindner-D’Addario M.; Sigman M. S.; Aspuru-Guzik A. A Comprehensive Discovery Platform for Organophosphorus Ligands for Catalysis. J. Am. Chem. Soc. 2022, 144 (3), 1205–1217. 10.1021/jacs.1c09718. 35020383

[ref26] Gensch T.; Smith S. R.; Colacot T. J.; Timsina Y. N.; Xu G.; Glasspoole B. W.; Sigman M. S. Design and Application of a Screening Set for Monophosphine Ligands in Cross-Coupling. ACS Catal. 2022, 12 (13), 7773–7780. 10.1021/acscatal.2c01970.

[ref27] Newman-Stonebraker S. H.; Smith S. R.; Borowski E.; Peters E.; Gensch T.; Johnson H. C.; Sigman M. S.; Doyle A. G. Univariate Classification of Phosphine Ligation State and Reactivity in Cross-Coupling Catalysis. Science 2021, 374 (6565), 301–308. 10.1126/science.abj4213. 34648340

[ref28] LeSueur A.; Tao N.; Doyle A.; Sigman M. Multi-Threshold Analysis for Chemical Space Mapping of Ni-Catalyzed Suzuki-Miyaura Couplings. Eur. J. Org Chem. 2024, 27 (36), e202400428 10.1002/ejoc.202400428.

[ref29] Balcells D.; Nova A. Designing Pd and Ni Catalysts for Cross-Coupling Reactions by Minimizing Off-Cycle Species. ACS Catal. 2018, 8 (4), 3499–3515. 10.1021/acscatal.8b00230.

[ref30] Vanni M.; Giulimondi V.; Ruiz-Ferrando A.; Krumeich F.; Clark A. H.; Mitchell S.; López N.; Pérez-Ramírez J. Selectivity Control in Palladium-Catalyzed CH_2_Br_2_ Hydrodebromination on Carbon-Based Materials by Nuclearity and Support Engineering. ACS Catal. 2023, 13 (9), 5828–5840. 10.1021/acscatal.2c06394.

[ref31] Kaiser S. K.; Fako E.; Surin I.; Krumeich F.; Kondratenko V. A.; Kondratenko E. V.; Clark A. H.; López N.; Pérez-Ramírez J. Performance Descriptors of Nanostructured Metal Catalysts for Acetylene Hydrochlorination. Nat. Nanotechnol. 2022, 17 (6), 606–612. 10.1038/s41565-022-01105-4. 35484211

[ref32] Kaiser S. K.; Fako E.; Manzocchi G.; Krumeich F.; Hauert R.; Clark A. H.; Safonova O. V.; López N.; Pérez-Ramírez J. Nanostructuring Unlocks High Performance of Platinum Single-Atom Catalysts for Stable Vinyl Chloride Production. Nat. Catal. 2020, 3 (4), 376–385. 10.1038/s41929-020-0431-3. 32292878 PMC7156288

[ref33] Di Liberto G.; Cipriano L. A.; Pacchioni G. Single Atom Catalysts: What Matters Most, the Active Site or The Surrounding?. ChemCatChem 2022, 14 (19), e202200611 10.1002/cctc.202200611.

[ref34] Falivene L.; Credendino R.; Poater A.; Petta A.; Serra L.; Oliva R.; Scarano V.; Cavallo L. SambVca 2. A Web Tool for Analyzing Catalytic Pockets with Topographic Steric Maps. Organometallics 2016, 35 (13), 2286–2293. 10.1021/acs.organomet.6b00371.

[ref35] Ravel B.; Newville M. ATHENA; ARTEMIS, HEPHAESTUS Data Analysis for X-Ray Absorption Spectroscopy Using IFEFFIT. J. Synchrotron Radiat. 2005, 12 (4), 537–541. 10.1107/S0909049505012719. 15968136

[ref36] Kresse G.; Furthmüller J. Efficiency of Ab-Initio Total Energy Calculations for Metals and Semiconductors Using a Plane-Wave Basis Set. Comput. Mater. Sci. 1996, 6 (1), 15–50. 10.1016/0927-0256(96)00008-0.

[ref37] Kresse G.; Furthmüller J. Efficient Iterative Schemes for Ab Initio Total-Energy Calculations Using a Plane-Wave Basis Set. Phys. Rev. B:Condens. Matter Mater. Phys. 1996, 54 (16), 11169–11186. 10.1103/PhysRevB.54.11169. 9984901

[ref38] Perdew J. P.; Burke K.; Ernzerhof M. Generalized Gradient Approximation Made Simple. Phys. Rev. Lett. 1996, 77 (18), 3865–3868. 10.1103/PhysRevLett.77.3865. 10062328

[ref39] Grimme S.; Antony J.; Ehrlich S.; Krieg H. A Consistent and Accurate Ab Initio Parametrization of Density Functional Dispersion Correction (DFT-D) for the 94 Elements H-Pu. J. Chem. Phys. 2010, 132 (15), 154104 10.1063/1.3382344. 20423165

[ref40] Blöchl P. E. Projector Augmented-Wave Method. Phys. Rev. B:Condens. Matter Mater. Phys. 1994, 50 (24), 17953–17979. 10.1103/PhysRevB.50.17953. 9976227

[ref41] Pracht P.; Bohle F.; Grimme S. Automated Exploration of the Low-Energy Chemical Space with Fast Quantum Chemical Methods. Phys. Chem. Chem. Phys. 2020, 22 (14), 7169–7192. 10.1039/C9CP06869D. 32073075

[ref42] Bannwarth C.; Ehlert S.; Grimme S. GFN2-XTB—An Accurate and Broadly Parametrized Self-Consistent Tight-Binding Quantum Chemical Method with Multipole Electrostatics and Density-Dependent Dispersion Contributions. J. Chem. Theory Comput. 2019, 15 (3), 1652–1671. 10.1021/acs.jctc.8b01176. 30741547

[ref43] Becke A. D. Density-Functional Thermochemistry. III. The Role of Exact Exchange. J. Chem. Phys. 1993, 98 (7), 5648–5652. 10.1063/1.464913.

[ref44] Miehlich B.; Savin A.; Stoll H.; Preuss H. Results Obtained with the Correlation Energy Density Functionals of Becke and Lee, Yang and Parr. Chem. Phys. Lett. 1989, 157 (3), 200–206. 10.1016/0009-2614(89)87234-3.

[ref45] Lee C.; Yang W.; Parr R. G. Development of the Colle-Salvetti Correlation-Energy Formula into a Functional of the Electron Density. Phys. Rev. B:Condens. Matter Mater. Phys. 1988, 37 (2), 785–789. 10.1103/PhysRevB.37.785. 9944570

[ref46] Frisch M. J.; Trucks G. W.; Schlegel H. B.; Scuseria G. E.; Robb M. A.; Cheeseman J. R.; Scalmani G.; Barone V.; Petersson G. A.; Nakatsuji H.; Li X.; Caricato M.; Marenich A. V.; Bloino J.; Janesko B. G.; Gomperts R.; Mennucci B.; Hratchian H. P.; Ortiz J. V.; Izmaylov A. F., Gaussian 16, Revision B.01.; Gaussian Inc.: Wallingford, CT, 2016.

[ref47] Poier D.; Usteri M. E.; Stoian D.; Mitchell S.; Marti R.; Pérez-Ramírez J. Experimental Data - Phosphine Ligand Reactivity Descriptors for Sonogashira-Hagihara Coupling over a Pd Single-Atom Catalyst; Zenodo, 2024.

[ref48] Loveday O. Computational Data - Phosphine Ligand Reactivity Descriptors for Sonogashira-Hagihara Coupling over a Pd Single-Atom Catalyst; ioChem-BD, 2023. 10.19061/iochem-bd-1-344

