# Supplementary material for: Ligand-Induced Activation of Single-Atom Palladium Heterogeneous Catalysts for Cross-Coupling Reactions
Source: ACS Nano. 2025 Jan 2;19(1):1424–32. doi: 10.1021/acsnano.4c14131 (PMC11752494; doi:10.1021/acsnano.4c14131)
Supplement: Supplementary file 1 — nn4c14131_si_001.pdf [file nn4c14131_si_001.pdf]

Supporting Information

Ligand-Induced Activation of Single-Atom Palladium Heterogeneous Catalysts for Cross-Coupling Reactions

Dario Poier[a], Oliver Loveday[b,c], Marc Eduard Usteri[d], Dragos Stoian[e], Núria López[b], Sharon Mitchell*[d], Roger Marti*[a], Javier Pérez-Ramírez*[d]*

[a] Institute of Chemical Technology, Haute École d'Ingénierie et d'Architecture Fribourg, HES-SO University of Applied Sciences and Arts Western Switzerland, 1700 Fribourg, Switzerland.

[b] Institute of Chemical Research of Catalonia (ICIQ-CERCA), The Barcelona Institute of Science and Technology (BIST), 43007 Tarragona, Spain.

[c] Department of Physical and Inorganic Chemistry, Universitat Rovira i Virgili, 43007 Tarragona, Spain.

[d] Institute for Chemical and Bioengineering, Department of Chemistry and Applied Biosciences, ETH Zurich, 8093 Zurich, Switzerland.

[e] Swiss Norwegian beamlines, European Synchrotron Radiation Facility, 38043 Grenoble, France.

* Corresponding authors

Núria López,	E-mail: nlopez@iciq.es
Sharon Mitchell,	E-mail: sharon.mitchell@chem.ethz.ch
Roger Marti,	E-mail: roger.marti@hefr.ch
Javier Pérez-Ramírez,	E-mail: jpr@chem.ethz.ch

Table of Contents

Abbreviations	3
Note S1. Catalyst Washing Procedure and Rationale	4
Performance Data of SAC-Phosphine Systems	6
Density Functional Theory Simulation Results	8
Pd_1@NC Performances and Phosphine Properties	13
Free Phosphine Structures	20
Geometries of SAC and SAC-Phosphine Systems	22
Topographic Heatmaps of Pd_{Pyrr3}–Phosphine Configurations	26
Geometries of Iodobenzene–SAC–Phosphine Configurations	28
Sonogashira-Hagihara Cross-Coupling Mechanism	29
References	30

Abbreviations

**General**	
GS	Graphitic support
KID	KRAKEN identifier number
Pyr_{2+2}	Square planar dipyridinic-dipyrrolic (2×N6+2×N5)
$Pyri_4$	Square planar tetrapyridinic (4×N6)
$Pyrr_3$	Trigonal planar tripyrrolic (3×N5)
SAC	Single-atom catalyst
SH	Sonogashira-Hagihara
TOF	Turnover frequency
TON	Turnover number
**Techniques**	
AC-ADF	Aberration corrected annular dark-field
DFT	Density functional theory
EDX	Energy dispersive X-ray spectroscopy
GC-FID	Gas chromatography flame ionization detection
HAADF	High-angle annular dark-field detection
ICP-OES	Inductively coupled plasma optical emission spectroscopy
NMR	Nuclear magnetic resonance spectroscopy
STEM	Scanning transmission electron microscopy
**Compounds**	
AC	Activated carbon
Bippy	1′,3′,5′-Triphenyl-1′H-[1,4′]bipyrazol
CJP	CyJohnPhos, [1,1'-biphenyl]-2-yldicyclohexylphosphane
DI water	Deionized water
JP	JohnPhos, [1,1'-biphenyl]-2-yldi-tert-butylphosphane
MeCN	Acetonitrile
NC	Nitrogen-doped carbon
NEt_3	Triethylamine
PCy_3	Tricyclohexylphosphine
PPh_3	Triphenylphosphine
P^tBu_3	Tri-*tert*-butylphosphine
PTFE	Polytetrafluoroethylene

Note S1. Catalyst Washing Procedure and Rationale. The as-prepared Pd_1@NC catalyst was subjected to a multistep washing treatment to ensure that the observed catalytic activity originated from surface-bound palladium centers by removing weakly adsorbed Pd species that might otherwise leach into the reaction mixture. The procedure exploits the coordinating character of ethylene glycol to solubilize physisorbed metal atoms and avoid their redeposition, using a combination of KO^tBu and 1,4,7,10,13,16-hexaoxacyclooctadecane (18-Crown-6) to enhance the Pd-glycol interaction. Lastly, the material was washed with DI water and MeCN, to remove any remaining metal species and impurities.

Procedure: The reagents for this procedure were purchased at Chemie Brunschwig AG and used without further purification. All glass vessels were cleaned using aqua regia and water before use. The as-prepared Pd_1@NC was treated in 4 sequential steps (i–iv):

i) Ethylene glycol (55.6 g, 89.6 mmol) was added to the as-prepared Pd_1@NC (4.58 g) and stirred in a closed vessel at 363 K for 2 h. The solid was filtered from the hot solution and washed with hot (363 K) ethylene glycol (2×75 cm^3). Repetition of this step yielded i-Pd_1@NC.

ii) i-Pd_1@NC was added to a solution of $KO^{t}Bu$ (2.15 g, 19.2 mmol) and 18-Crown-6 (5.10 g, 19.3 mmol) in ethylene glycol (50.0 g, 80.5 mmol), and stirred in a closed vessel at 363 K for 2 h. The solid was filtered from the hot solution and washed with hot (363 K) ethylene glycol (2×75 cm^3) and DI water (2×75 cm^3). Repetition of this step yielded ii-Pd_1@NC.

iii) ii-Pd_1@NC was combined with DI water (50 g, 2.78 mol) and stirred in a closed vessel at 363 K for 2 h. The solid was then filtered from the hot solution and washed with hot

(363 K) water (2×75 cm^3) and MeCN (2×75 cm^3). Repetition of this step yielded iii-Pd_1@NC.

iv) iii-Pd_1@NC was stirred in a MeCN:NEt_3 solution (4:1 v/v, 38.7 g) in a closed vessel at 363 K for 2 h, filtered from the hot solution, and washed with hot (363 K) MeCN:NEt_3 (4:1 v/v, 2×75 cm^3) and MeCN (2×75 cm^3). Repetition of this step yielded iv-Pd_1@NC, which was dried at 373 K for 48 h to obtain washed-Pd_1@NC (4.31 g) and stored under inert conditions at 293–303 K.

The conditions were chosen to avoid temperature-induced metal loss leading to metal nanoparticle formation, observed in attempts at higher temperatures while being 15 K higher than the reaction temperature. ICP-OES confirmed a reduction in metal loading from 0.53 wt % to 0.22 wt %, verifying the effectiveness of the washing procedure.

Performance Data of SAC-Phosphine Systems

Table S1. Yield and turnover frequency (TOF) evidenced in the Sonogashira-Hagihara (SH) coupling for the studied SAC-phosphine systems. Listed in ascending order according to the KRAKEN identifier number (KID) of the phosphine.

KID	Name	Phosphine class	Yield[a] / %	t_{100} / h	TOF[b] / h^{-1}
**1**	XPhos	CyJohnPhos	30	1.21	83
**3**	SPhos	CyJohnPhos	46	$7.41 \cdot 10^{-1}$	135
**4**	RuPhos	CyJohnPhos	31	1.50	67
**5**	CyJohnPhos	CyJohnPhos	73	$3.10 \cdot 10^{-2}$	3223
**8**	P^tBu_3	$Alkyl_3$	23	1.88	53
**9**	P(*o*-Tol)$_3$	$Aryl_3$	67	$1.26 \cdot 10^{-1}$	793
**11**	PCy_3	$Alkyl_3$	14	5.30	19
**15**	P(2-OMe-Ph)$_3$	$Aryl_3$	35	$6.57 \cdot 10^{-1}$	152
**17**	PPh_3	$Aryl_3$	81	$2.13 \cdot 10^{-5}$	4687406
**42**	JohnPhos	JohnPhos	16	4.52	22
**54**	P(2-Furyl)$_3$	$Aryl_3$	85	$8.22 \cdot 10^{-7}$	121618542
**62**	P(*p*-OMe-Ph)$_3$	$Aryl_3$	74	$3.04 \cdot 10^{-3}$	32879
**68**	PCy_2Ph	Miscellaneous	45	$6.17 \cdot 10^{-1}$	162
**79**	P^iPr_3	$Alkyl_3$	17	2.99	33
**84**	P(allyl)Ph_2	Miscellaneous	60	$8.33 \cdot 10^{-3}$	12001
**89**	^{t}BuBrettPhos	JohnPhos	9	$3.51 \cdot 10^{1}$	3
**90**	^{t}BuXPhos	JohnPhos	10	$1.11 \cdot 10^{1}$	9
**102**	BrettPhos	CyJohnPhos	8	$3.64 \cdot 10^{1}$	3
**104**	JackiePhos	Miscellaneous	45	$2.69 \cdot 10^{-1}$	372
**139**	P(OEt)Ph_2	N,O-substituent	76	$6.73 \cdot 10^{-3}$	14866
**148**	P(OEt)$_3$	N,O-substituent	54	$2.00 \cdot 10^{-1}$	501
**217**	*o*-Tol_2Ph-Diazaphosphinane	P-heterocycle	4	$1.69 \cdot 10^{1}$	6
**239**	P(*n*Oct)$_3$	$Alkyl_3$	2	$1.38 \cdot 10^{1}$	7
**251**	P(allyl)$_3$	Miscellaneous	4	$4.45 \cdot 10^{1}$	2
**263**	DavePhos	CyJohnPhos	54	$5.66 \cdot 10^{-1}$	177
**277**	MEPHOS	CyJohnPhos	71	$2.58 \cdot 10^{-2}$	3874

KID	Name	Phosphine class	Yield[a,b] / %	t_{100} / h	TOF[b,c] / h^{-1}
**278**	PhDave-Phos	$Aryl_3$	67	$4.65 \cdot 10^{-2}$	2150
**280**	^{t}BuMePhos	JohnPhos	17	3.34	30
**281**	^{t}BuDavePhos	JohnPhos	15	5.33	19
**291**	$Me_4{}^t$BuXPhos	JohnPhos	13	$1.21 \cdot 10^{1}$	8
**327**	Cy-BippyPhos	Miscellaneous	68	$3.28 \cdot 10^{-3}$	30533
**329**	CM-Phos	Miscellaneous	16	6.84	15
**338**	PAd_2(2-piperidyl-Ph)	Miscellaneous	12	9.12	11
**340**	Ad-Bippyphos	Miscellaneous	15	4.50	22
**351**	EtAtaPhos	Miscellaneous	23	2.95	34
**401**	Azaphospha-bicycloheptane	P-heterocycle	10	6.80	15
**449**	$P(NEt_2)_3$	N,O-substituent	11	6.32	16
**458**	PPh_2(1-Isochinolin-2-Nap)	$Aryl_3$	51	$2.40 \cdot 10^{-1}$	416
**640**	Benzodioxa-phosphepine	P-heterocycle	41	$9.96 \cdot 10^{-1}$	100
**648**	$P(O^tBu)_2N(^iPr)_2$	N,O-substituent	15	4.97	20
**650**	$P(OBn)_2NEt_2$	N,O-substituent	59	$4.84 \cdot 10^{-2}$	2064
**-**	-	-	17	7.78	13

[a] Reaction conditions: iodobenzene (**1**, 1.6 mmol), ethynylbenzene (**2**, 2.3 mmol), triethylamine (NEt_3, 4.4 mmol), and MeCN (4.8 cm^3), 1,3,5-trimethylbenzene (0.25 mmol) palladium catalyst (0.53 wt % Pd, 0.2 mol %), phosphine (2.0 mol %) and copper(I) iodide (CuI, 4.0 mol %), 24 h at 353 K, Ar-atmosphere.

[b] Yield of 1,2-diphenylethyne (**3**) after 4 h, determined by GC-FID.

[c] Calculated by dividing the turnover number (TON) of 100 by the time (t_{100}) necessary for the system to reach it ($TOF = TON \cdot t^{-1} = 100 \cdot t_{100}{}^{-1}$). The specific t_{100} for each SAC-phosphine combination was estimated by monitoring the evolution of the 1,2-diphenylethyne (**3**) yield in the SH coupling (**Figures S1** and **S2**) and interpolating these data.

Density Functional Theory Simulation Results

Table S2. Adsorption energies (E_{ads}) of the most stable configurations for the phosphine–SAC systems at the square planar tetrapyridinic (4×N6, $Pyri_4$), dipyridinic-dipyrrolic (2×N6+2×N5, Pyr_{2+2}) and trigonal planar tripyrrolic (3×N5, $Pyrr_3$) cavities as well as graphitic support (GS, **Figure S10**). Taking into account the compensation through the entropic contribution, the comparison reveals that only $Pyrr_3$-stabilized Pd atoms allow for sufficiently strong adsorption of the phosphine.

Phosphine	$E_{ads,Pyri4}$[a] / eV	$E_{ads,Pyr2+2}$[b] / eV	$E_{ads,Pyrr3}$[c] / eV	$E_{ads,GS}$ / eV
PPh_3	−0.82	−0.86	−2.44	−0.83
P^tBu_3	−0.72	−0.73	−2.04	−0.70
PCy_3	−0.90	−0.91	−2.54	−0.86
JohnPhos	−0.88	−0.89	−1.30	−0.85
CyJohnPhos	−0.96	−0.94	−2.23	−0.96

[a] Configurations are shown in **Figure S11**.

[b] Configurations are shown in **Figure S12**.

[c] Configurations are shown in **Figure S13**.

Table S3. Bader charges (q_{Bader}) for P and Pd of the most stable Pd_{Pyrr3}–phosphine configurations and the ligand-free Pd_1@NC at the $Pyrr_3$ site (Pd_{Pyrr3}).

System	$q_{Bader,P}$ / -	$q_{Bader,Pd}$ / -
Pd_{Pyrr3}	-	0.72
Pd_{Pyrr3}–PPh_3	1.60	0.51
Pd_{Pyrr3}–P^tBu_3	0.82	0.55
Pd_{Pyrr3}–PCy_3	1.18	0.50
Pd_{Pyrr3}–JohnPhos	1.18	0.50
Pd_{Pyrr3}–CyJohnPhos	1.13	0.51

Table S4. Pd–P ($d_{Pd–P}$), Pd–carrier ($d_{Pd–carrier}$, measured perpendicular to the carrier plane), and Pd–N ($d_{Pd–N}$, anchoring nitrogen atoms of the cavity) distances of the most stable Pd_{Pyrr3}–phosphine configurations and the ligand-free Pd_{Pyrr3}.

System	$d_{Pd–P}$ / Å	$d_{Pd–carrier}$ / Å	$d_{Pd–N1}$ / Å	$d_{Pd–N2}$ / Å	$d_{Pd–N3}$ / Å
Pd_{Pyrr3}–PPh_3	2.22	1.25	2.11	2.23	2.05
Pd_{Pyrr3}–P^tBu_3	2.40	1.25	2.12	2.14	2.14
Pd_{Pyrr3}–PCy_3	2.28	1.27	2.09	2.01	2.33
Pd_{Pyrr3}–JohnPhos	2.34	1.33	2.25	2.08	2.23
Pd_{Pyrr3}–CyJohnPhos	2.28	1.27	2.20	2.04	2.17
Pd_{Pyrr3}	-	1.00	2.02	2.02	2.02

Table S5. Energy demand for the deformation (E_{def}) of the phosphine ligands from their equilibrium state upon coordination with Pd_{Pyrr3} atoms.

System	$E_{def,P}$ / eV	$E_{def,Pd}$ / eV
Pd_{Pyrr3}–PPh_3	0.27	0.24
Pd_{Pyrr3}–P^tBu_3	0.06	0.19
Pd_{Pyrr3}–PCy_3	0.29	0.35
Pd_{Pyrr3}–JohnPhos	1.06	0.45
Pd_{Pyrr3}–CyJohnPhos	0.61	0.28

Table S6. Percent buried volumes (%V_{bur}) within the Pd-phosphine and Pd-carrier hemisphere of the palladium-centered V_{bur} sphere (r = 3.5 Å, V = 179.6 Å^3) for the most stable Pd_{Pyrr3}–phosphine configurations, calculated for the whole hemisphere and its single quadrants.[2]

Hemisphere	%$V_{bur,total}$[a] / -	%$V_{bur,Q1}$[b] / -	%$V_{bur,Q2}$[b] / -	%$V_{bur,Q3}$[b] / -	%$V_{bur,Q4}$[b] / -
Pd_{Pyrr3}–PPh_3	47.7	54.3	47.0	45.9[d]	43.5[c,d]
$Pd_{Pyrr3,PPh3}$–carrier	63.2	-	-	-	-
Pd_{Pyrr3}–P^tBu_3	49.6	49.6[d]	49.5	50.4	49.0[c,d]
$Pd_{Pyrr3,P\mathit{t}Bu3}$–carrier	63.3	-	-	-	-
Pd_{Pyrr3}–PCy_3	48.9	50.7[d]	43.2[c,d]	53.1	48.4
$Pd_{Pyrr3,PCy3}$–carrier	63.3	-	-	-	-
Pd_{Pyrr3}–JohnPhos	49.0	55.7	39.3[c,d]	43.9[d]	57.0
$Pd_{Pyrr3,JP}$–carrier	62.0	-	-	-	-
Pd_{Pyrr3}–CyJohnPhos	48.6	54.4	40.9[c,d]	44.7[d]	54.4
$Pd_{Pyrr3,CJP}$–carrier	63.1	-	-	-	-

[a] Calculated by dividing the V_{bur} by the hemisphere volume ($V_{sphere,Vbur}$×0.5) of the V_{bur} sphere and multiplying it by 100.

[b] Calculated by dividing the V_{bur} of the phosphine within quadrant Q_x (x = 1, 2, 3 or 4) by the quadrant volume ($V_{sphere,Vbur}$×0.125) of the V_{bur} sphere and multiplying it by 100.

[c] Quadrant with the lowest volume occupied by the phosphine (Q_{min}).

[d] Pair of adjacent quadrants which on average exhibit the lowest volume occupied by the phosphine ($Q_{adj,min}$).

Table S7. %V_{bur} within the Pd-phosphine and Pd-carrier hemisphere of the palladium-centered V_{bur} sphere (r = 4.5 Å, V = 381.7 Å^3) for the most stable Pd_{Pyrr3}–phosphine configurations, calculated for the whole hemisphere and its single quadrants.[2]

**Hemisphere**	**%$V_{bur,total}$[a] / -**	**%$V_{bur,Q1}$[b] / -**	**%$V_{bur,Q2}$[b] / -**	**%$V_{bur,Q3}$[b] / -**	**%$V_{bur,Q4}$[b] / -**
Pd_{Pyrr3}–PPh_3	39.8	47.4	41.7	39.1	30.8
Pd_{Pyrr3}–P^tBu_3	42.3	40.6	39.9	45.1	43.7
Pd_{Pyrr3}–PCy_3	42.3	42.2	35.7	49.1	42.1
Pd_{Pyrr3}–JohnPhos	43.3	52.1	32.7	35.8	52.7
Pd_{Pyrr3}–CyJohnPhos	42.3	50.6	32.1	37.1	49.2

[a] Calculated by dividing the V_{bur} by the hemisphere volume ($V_{sphere,Vbur}$×0.5) of the V_{bur} sphere and multiplying it by 100.

[b] Calculated by dividing the V_{bur} of the phosphine within quadrant Q_x (x = 1, 2, 3 or 4) by the quadrant volume ($V_{sphere,Vbur}$×0.125) of the V_{bur} sphere and multiplying it by 100.

Table S8. %V_{bur} within the Pd-phosphine and Pd-carrier hemisphere of the palladium-centered V_{bur} sphere (r = 6.5 Å, V = 1150.4 Å^3) for the most stable Pd_{Pyrr3}–phosphine configurations, calculated for the whole hemisphere and its single quadrants.[2]

**Hemisphere**	**%$V_{bur,total}$[a] / -**	**%$V_{bur,Q1}$[b] / -**	**%$V_{bur,Q2}$[b] / -**	**%$V_{bur,Q3}$[b] / -**	**%$V_{bur,Q4}$[b] / -**
Pd_{Pyrr3}–PPh_3	25.1	35.1	26	22	17.5
Pd_{Pyrr3}–P^tBu_3	25.4	22.7	20.8	29.5	28.6
Pd_{Pyrr3}–PCy_3	28.2	23.8	25.4	35.5	27.9
Pd_{Pyrr3}–JohnPhos	29.9	38.3	17.5	20	43.9
Pd_{Pyrr3}–CyJohnPhos	31.2	37.7	19.1	27.4	40.5

[a] Calculated by dividing the V_{bur} by the hemisphere volume ($V_{sphere,Vbur}$×0.5) of the V_{bur} sphere and multiplying it by 100.

[b] Calculated by dividing the V_{bur} of the phosphine within quadrant Q_x (x = 1, 2, 3 or 4) by the quadrant volume ($V_{sphere,Vbur}$×0.125) of the V_{bur} sphere and multiplying it by 100.

Table S9. Comparison of the $d_{Pd–P}$, and $d_{Pd–N}$ distances before and during the adsorption of iodobenzene **1** at the metal center for the PPh_3 and CJP systems.

**System**	$d_{Pd–P}$ **/ Å**	$d_{Pd–N1}$ **/ Å**	$d_{Pd–N2}$ **/ Å**	$d_{Pd–N3}$ **/ Å**
Pd_{Pyrr3}–PPh_3	2.22	2.11	2.23	2.05
IPh–Pd_{Pyrr3}–PPh_3[a]	2.56	2.22	2.16	2.17
Pd_{Pyrr3}–CyJohnPhos	2.28	2.20	2.04	2.17
IPh–Pd_{Pyrr3}–CyJohnPhos[a]	2.39	2.51	2.14	2.12

[a] Configurations are shown in **Figure S16**.

Pd_1@NC Performances and Phosphine Properties

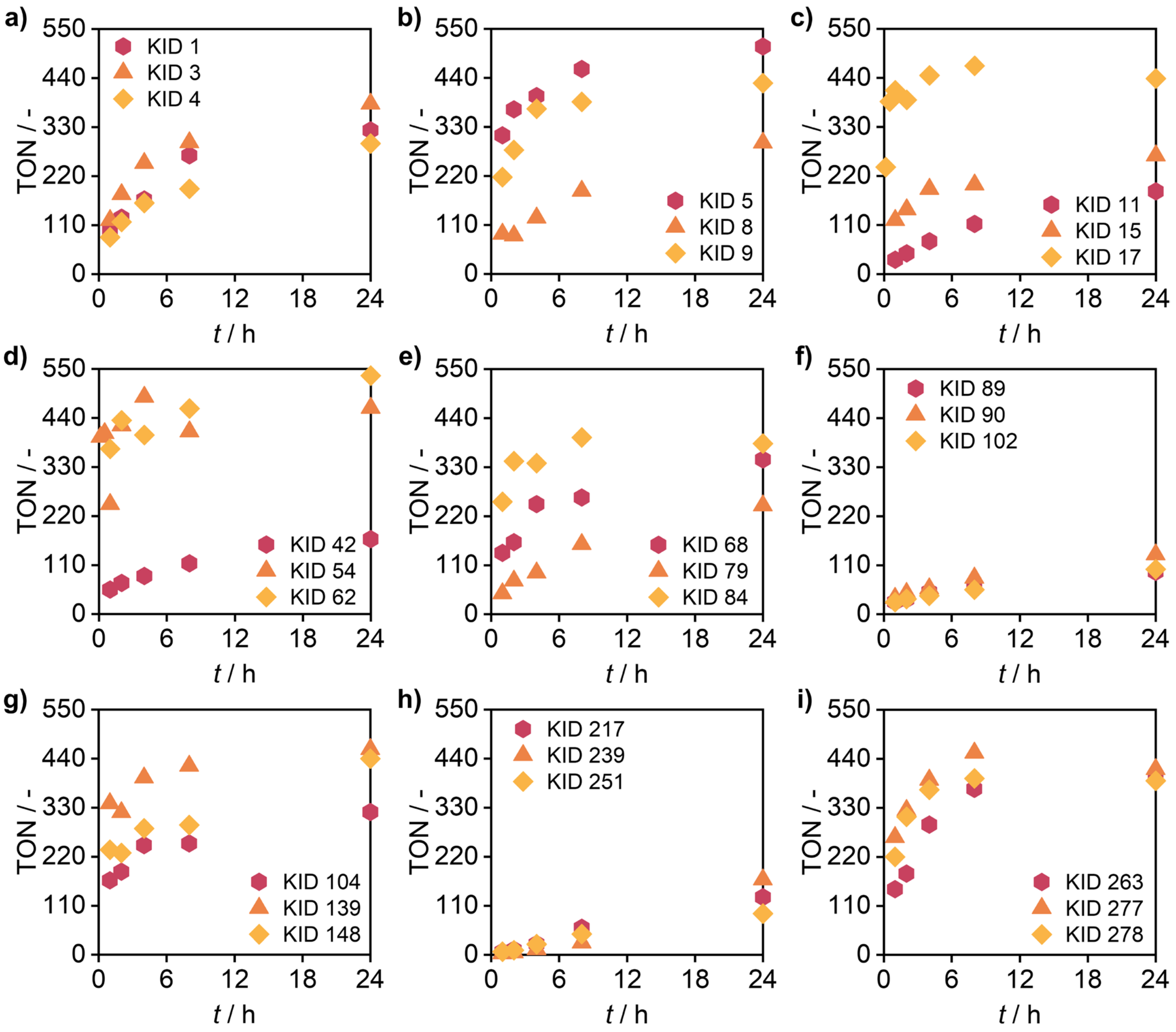

Figure S1. a–i) TON for 1,2-diphenylethyne (**3**) production in the SH coupling at 353 K over selected SAC-phosphine systems, using the as-prepared Pd_1@NC. The respective ligands are identified inset by their KID (**Table S1**). Reaction conditions: iodobenzene (**1**, 1.6 mmol), ethynylbenzene (**2**, 2.3 mmol), triethylamine (NEt_3, 4.4 mmol), and MeCN (4.8 cm^3), 1,3,5-trimethylbenzene (0.25 mmol) palladium catalyst (0.53 wt % Pd, 0.2 mol %), phosphine (2.0 mol %) and copper(I) iodide (CuI, 4.0 mol %), 24 h at 353 K, Ar-atmosphere.

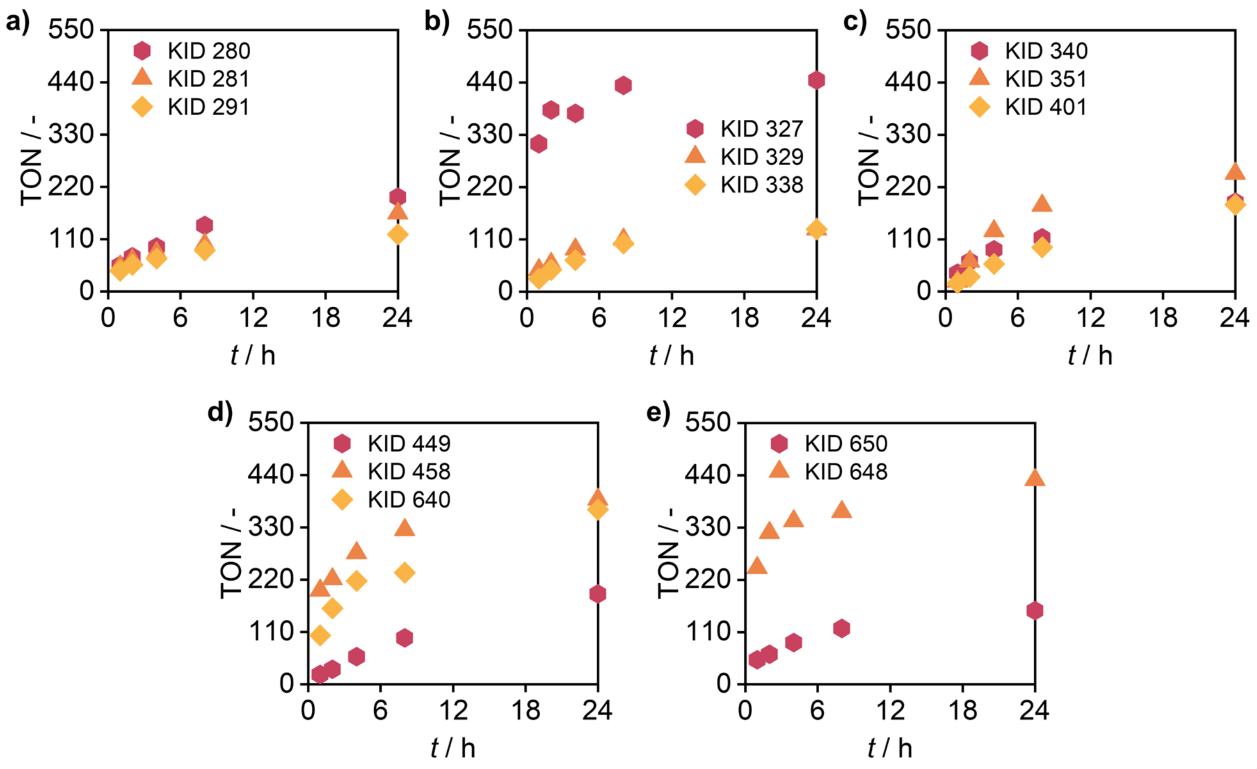

Figure S2. a–e) TON for 1,2-diphenylethyne (**3**) production in the SH coupling at 353 K over the selected SAC-phosphine systems, using the as-prepared Pd_1@NC. The respective ligands are identified inset by their KID (**Table S1**). Reaction conditions: iodobenzene (**1**, 1.6 mmol), ethynylbenzene (**2**, 2.3 mmol), triethylamine (NEt_3, 4.4 mmol), and MeCN (4.8 cm^3), 1,3,5-trimethylbenzene (0.25 mmol) palladium catalyst (0.53 wt % Pd, 0.2 mol %), phosphine (2.0 mol %) and copper(I) iodide (CuI, 4.0 mol %), 24 h at 353 K, Ar-atmosphere.

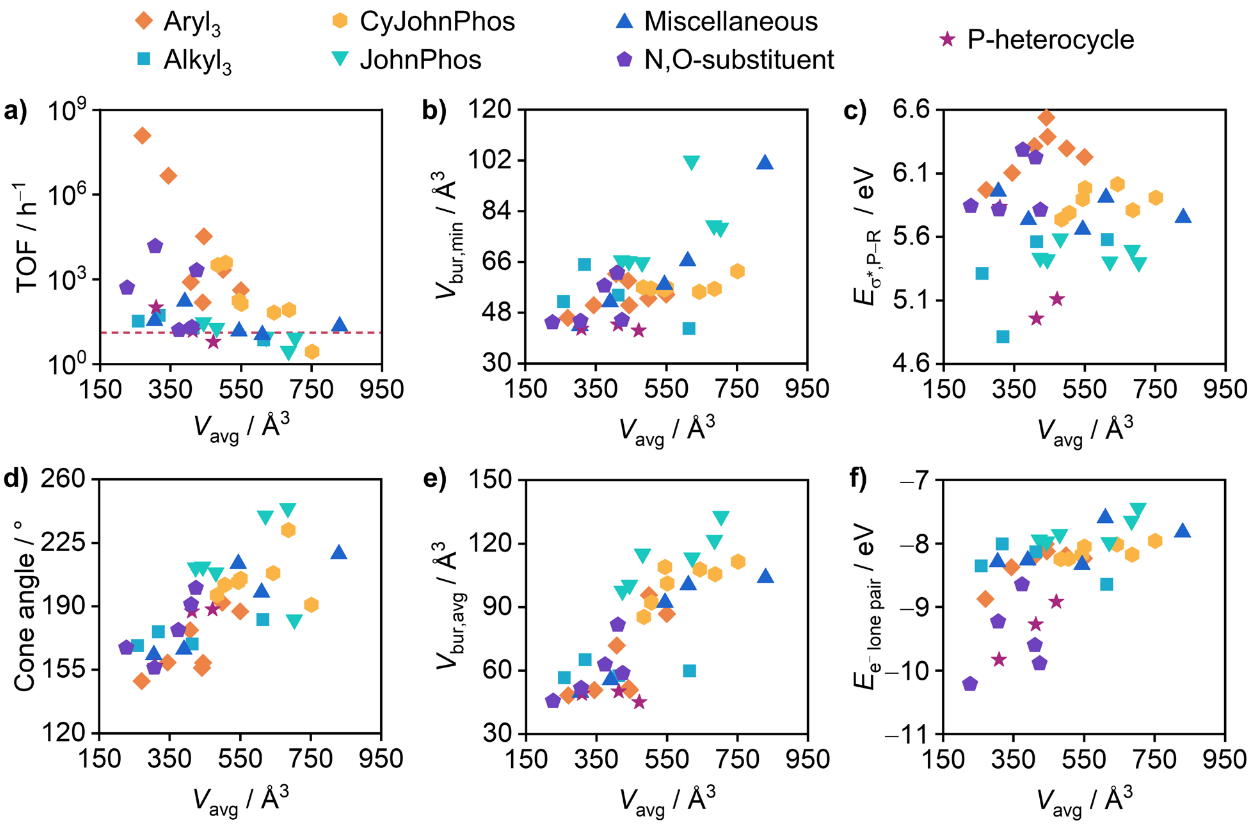

Figure S3. a) Comparison of the TOF for 1,2-diphenylethyne (**3**) production in the SH coupling at 353 K over the SAC-phosphine systems, using the as-prepared Pd_1@NC versus the average conformer volume of the free phosphines. The dashed red line shows the activity in the absence of phosphine. Comparison of **b)** the minimum buried volume in an organometallic complex, **c)** the average energy of the antibonding P–R orbital, **d)** cone angle, **e)** average buried volume in an organometallic complex, and **f)** electron lone pair energy of the tested phosphines plotted versus the average conformer volume of the phosphines. Values for the ligand properties were obtained from the KRAKEN database.[3]

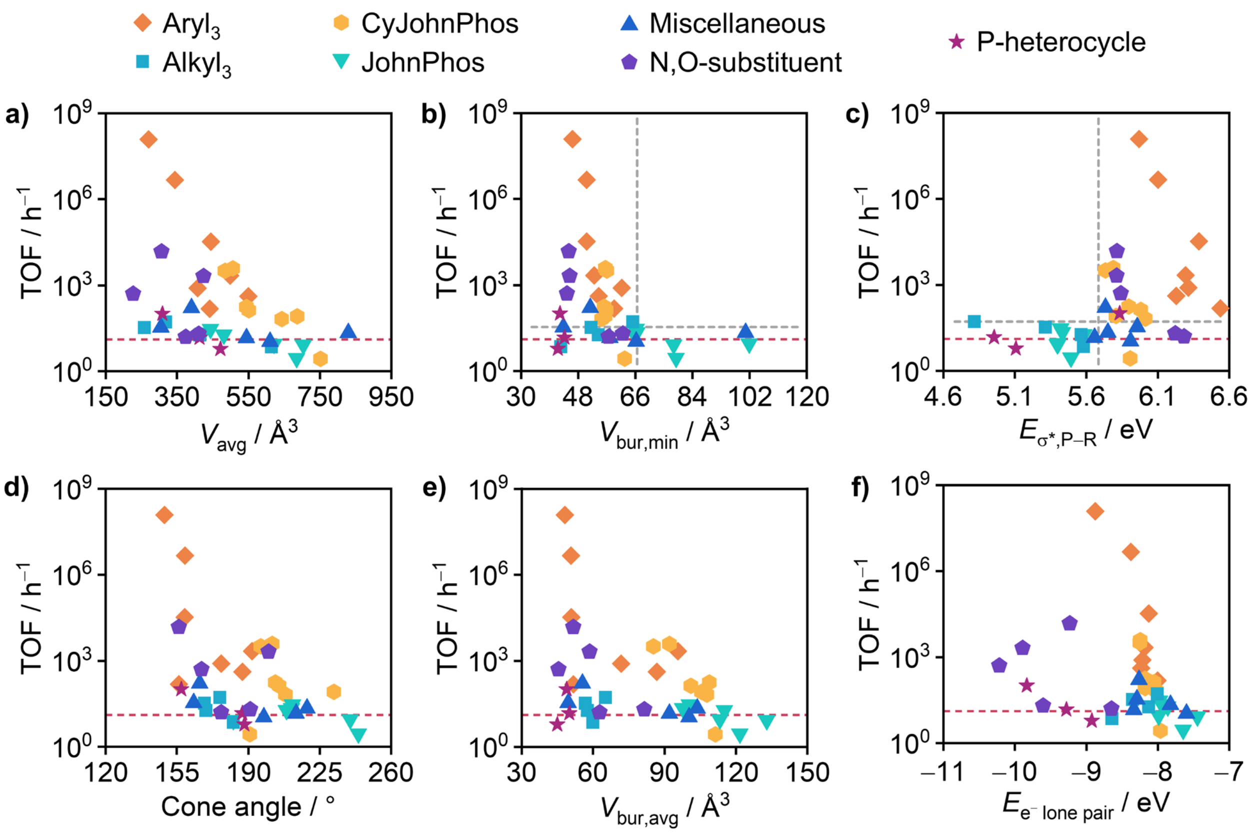

Figure S4. Comparison of the TOF for 1,2-diphenylethyne (**3**) production in the SH coupling at 353 K over the SAC-phosphine systems, using the as-prepared Pd_1@NC, versus the **a)** average conformer volume of the free phosphines, **b)** minimum buried volume in an organometallic complex, **c)** average energy of the antibonding P–R orbital, **d)** cone angle, **e)** average buried volume in an organometallic complex, and **f)** electron lone pair energy. Values for the ligand properties were obtained from the KRAKEN database.[3] The dashed, red line represents activity in the absence of phosphine; the dashed, gray line represents apparent reactivity zones, similar to those identified in studies concerning organometallic complexes.[4,5]

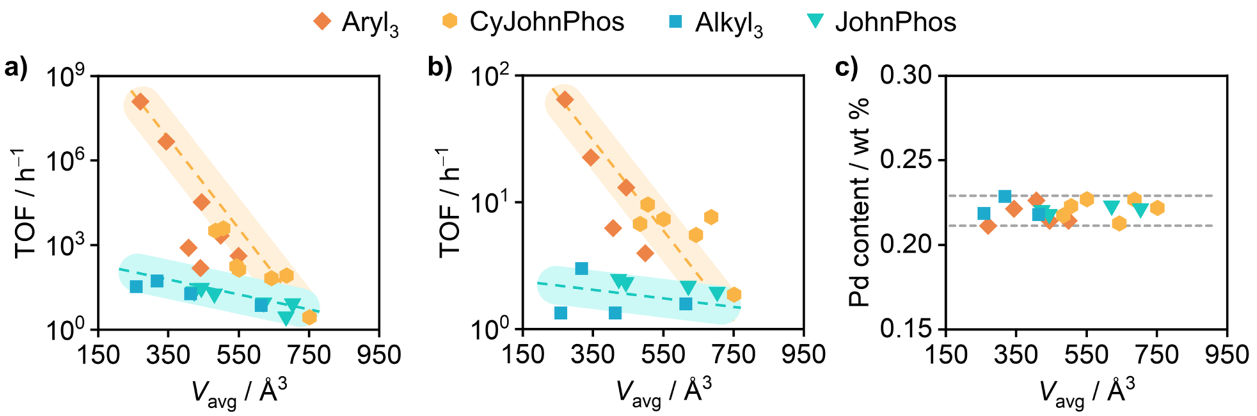

Figure S5. Comparison of the TOF for 1,2-diphenylethyne (**3**) production in the SH coupling at 353 K over the SAC-phosphine systems, using **a)** the as-prepared Pd_1@NC (reaction temperature of 353 K) and **b)** the washed-Pd_1@NC (reaction temperature of 348 K), versus the average conformer volume of the free phosphines. TOF for the washed-Pd_1@NC in b) was determined after its second application in the SH coupling (Run 2, **Figures S6** and **S7**). The impact of reaction temperature on the system has been addressed in previous work.[6] **c)** Palladium content of the wahsed-Pd_1@NC after two uses in the SH coupling as described for b).

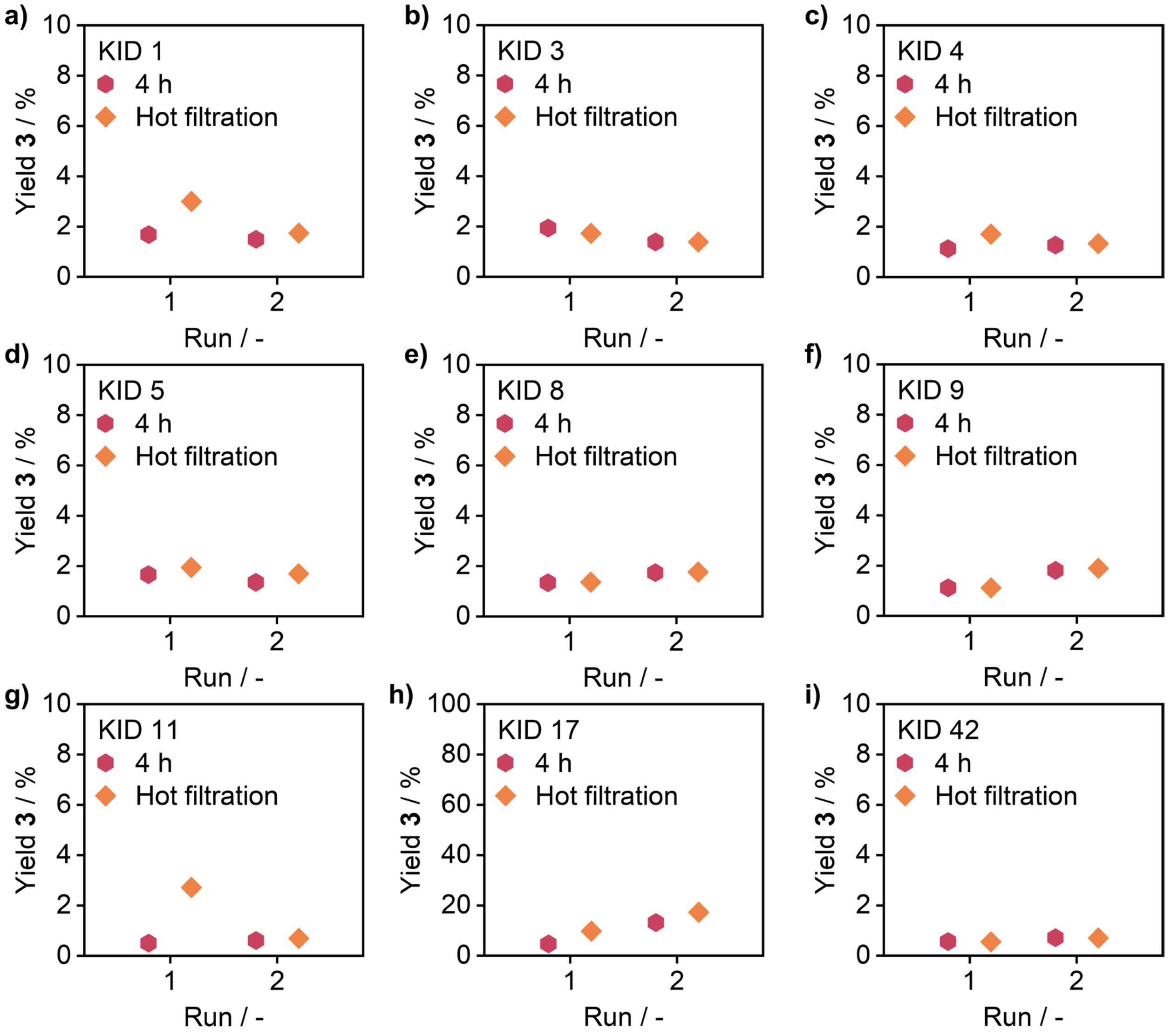

Figure S6. a–i) Yield of 1,2-diphenylethyne (**3**) after 4 h at 348 K in the presence of selected SAC-phosphine systems, using the washed-Pd_1@NC in two consecutive batch runs. After 4 h the catalyst was separated from the reaction by transferring the hot reaction mixture into a syringe and filtering it using a PTFE syringe filter. After taking a 4 h-aliquot for analysis (4 h), the solution was continued to stir for an additional 4 h (Hot filtration) without catalyst at 348 K. The respective phosphine is identified by their KID (**Table S1**).

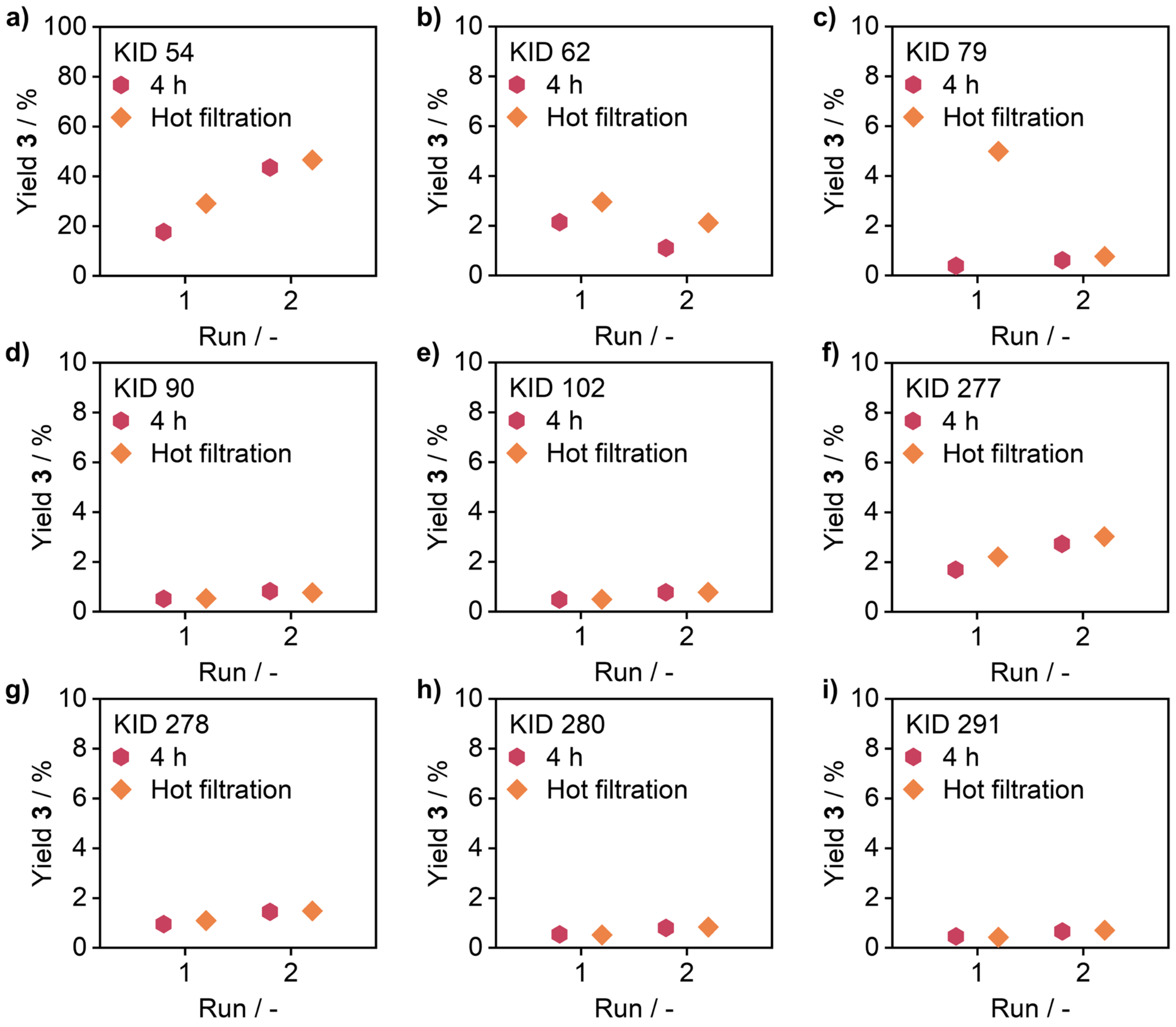

Figure S7. a)–i) Yield of 1,2-diphenylethyne (**3**) after 4 h at 348 K in the presence of selected SAC-phosphine systems, using the washed-Pd_1@NC in two consecutive batch runs. After 4 h the catalyst was separated from the reaction by transferring the hot reaction mixture into a syringe and filtering it using a PTFE syringe filter. After taking a 4 h-aliquot for analysis (4 h), the solution was continued to stir for an additional 4 h (Hot filtration) without catalyst at 348 K. The respective phosphine is identified by their KID (**Table S1**).

Free Phosphine Structures

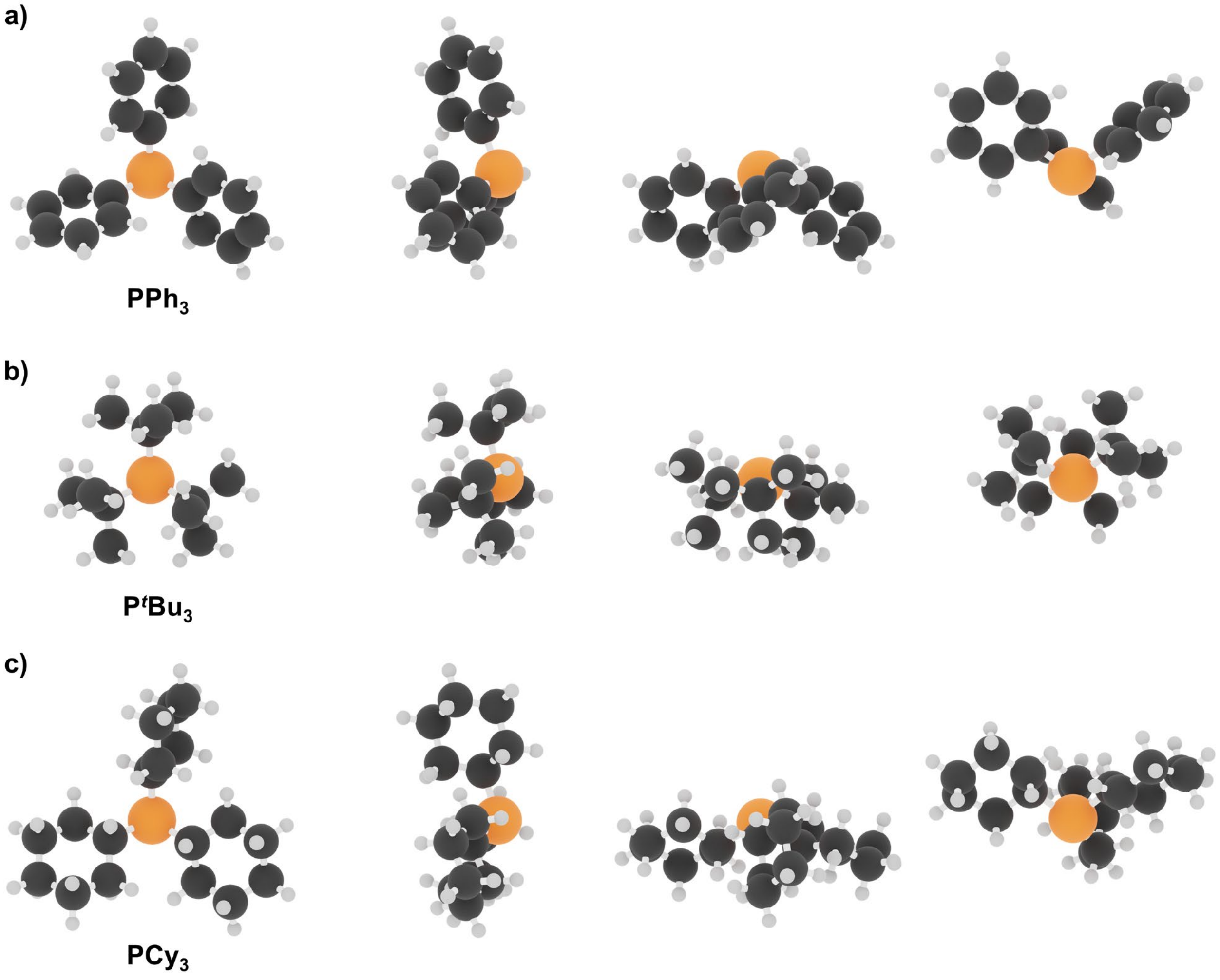

Figure S8. Comparative geometry of the free **a)** PPh_3, **b)** P^tBu_3, and **c)** PCy_3 phosphine ligands (hydrogen: white, carbon: dark gray, phosphorous: orange). Left-to-right: the front (phosphorous electron lone pair facing to the back), the right (90° rotation), top-down and bottom-up view.

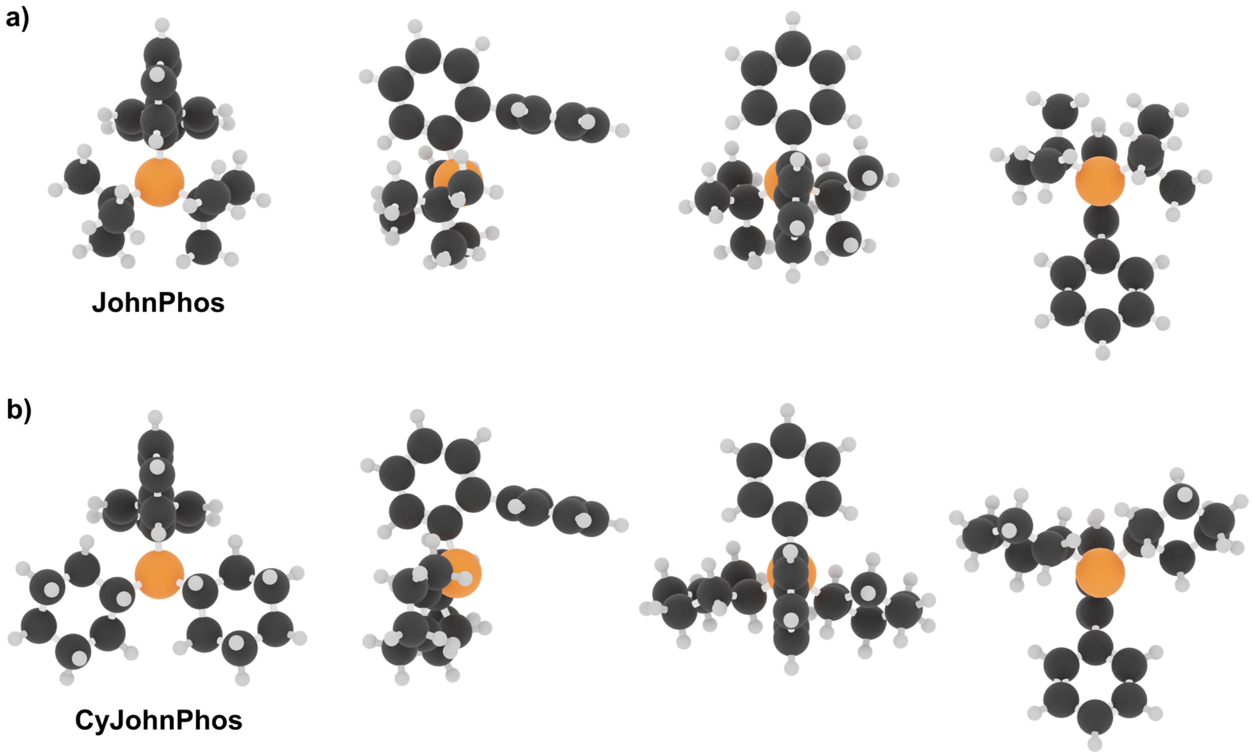

Figure S9. Comparative geometry of the free **a)** JP and **b)** CJP phosphine ligands (hydrogen: white, carbon: dark gray, phosphorous: orange). Left-to-right: the front (phosphorous electron lone pair facing to the back), the right (90° rotation), top-down and bottom-up view.

Geometries of SAC and SAC-Phosphine Systems

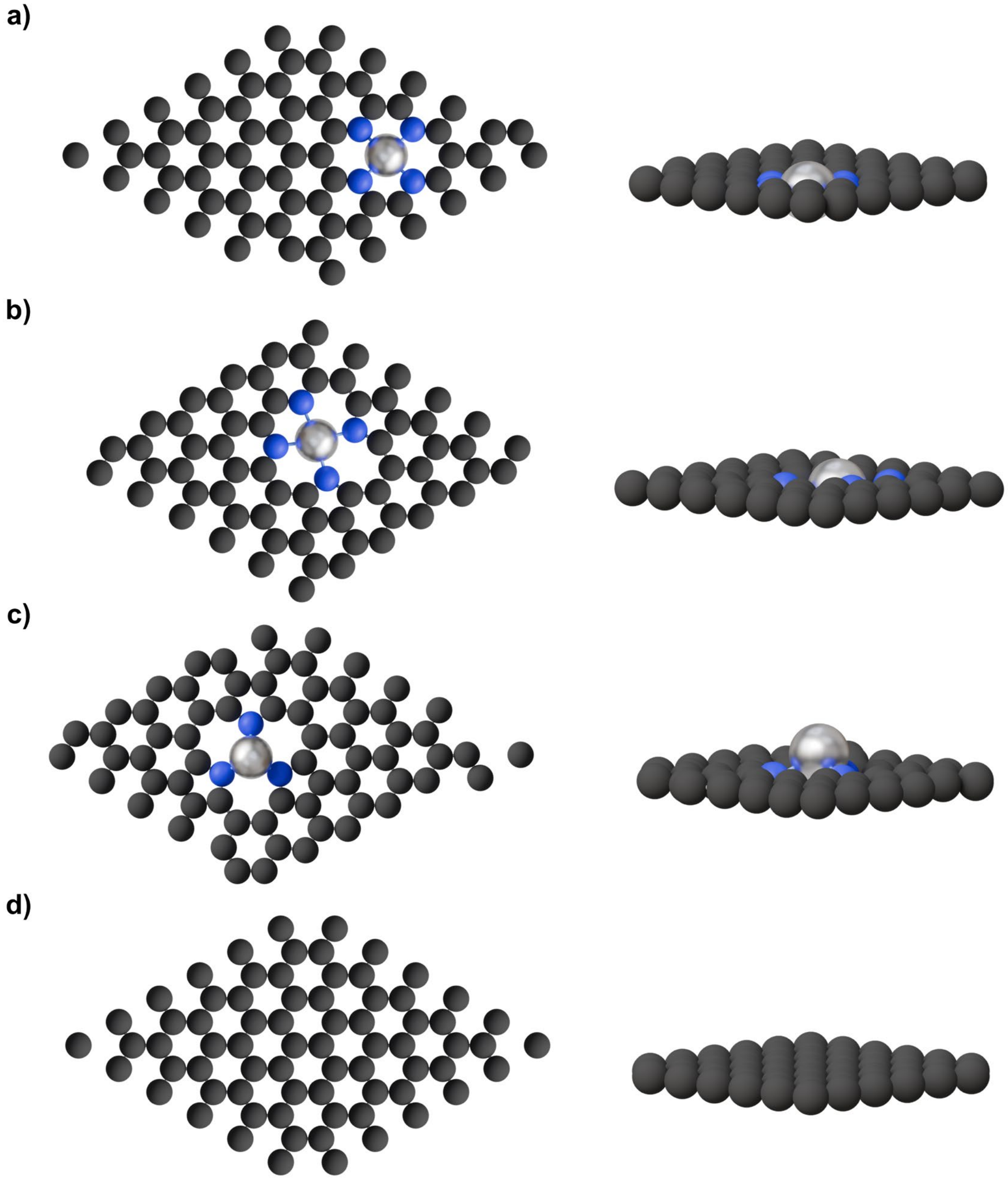

Figure S10. Geometries of the DFT-optimized Pd–SAC structures with the palladium atoms stabilized in the **a)** Pyri$_4$, **b)** Pyr$_{2+2}$, or **c)** Pyrr$_3$ cavity (carbon: dark gray, nitrogen: blue, palladium: silver). The **d)** nitrogen-free GS is used as a reference for the adsorption of the phosphine on the support.

a)

b)

c)

d)

e)

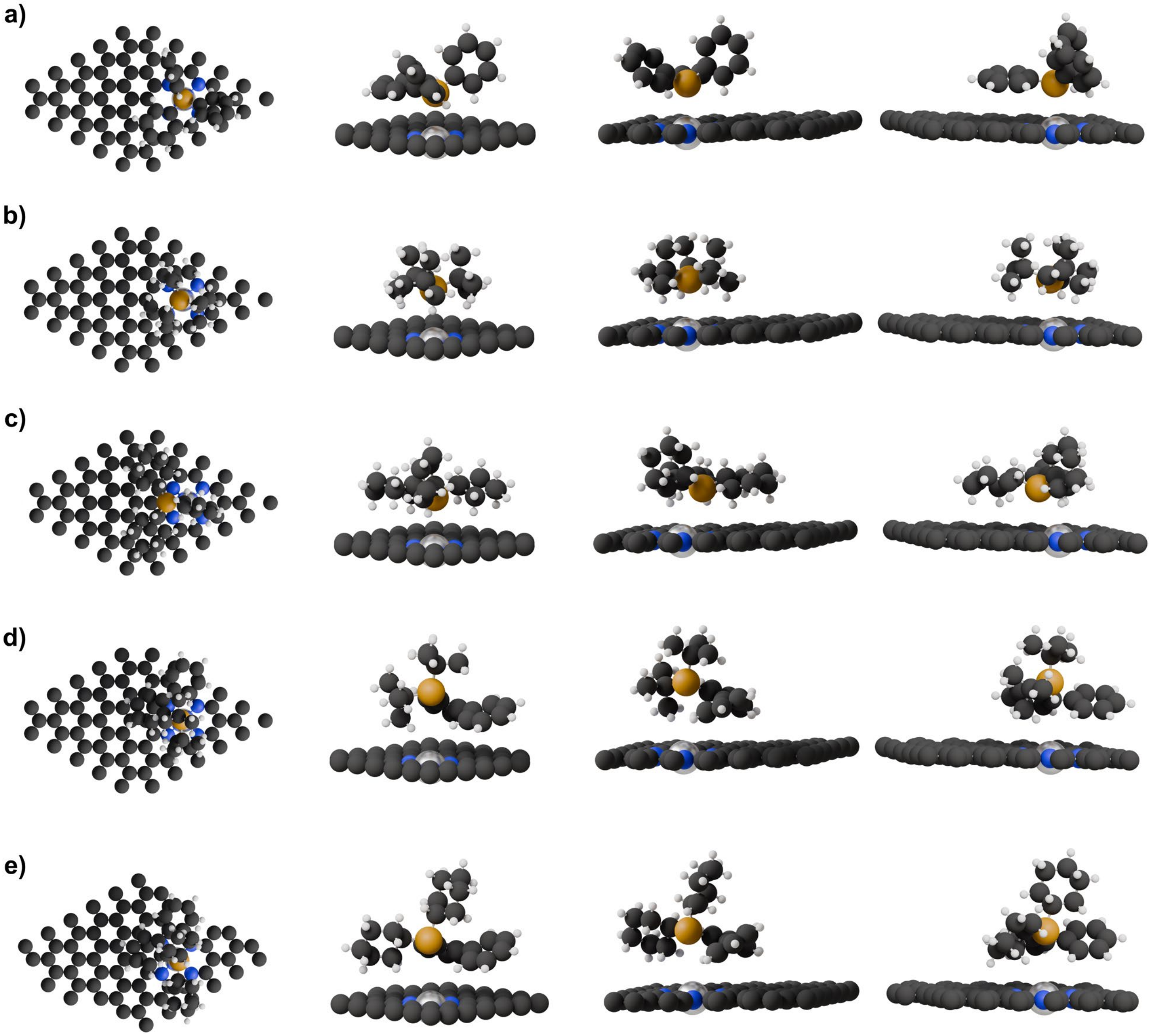

Figure S11. Most stable DFT-optimized phosphine–SAC configurations at the $Pyri_4$ cavity for **a)** PPh_3, **b)** P^tBu_3, **c)** PCy_3, **d)** JP, and **e)** CJP (hydrogen: white, carbon: dark gray, nitrogen: blue, phosphorous: orange, palladium: silver). Based on the E_{ads} for this square planar in-plane configuration, which equals the adsorption of the phosphine on the GS, the palladium center is considered to remain inactive for interaction with the phosphine.

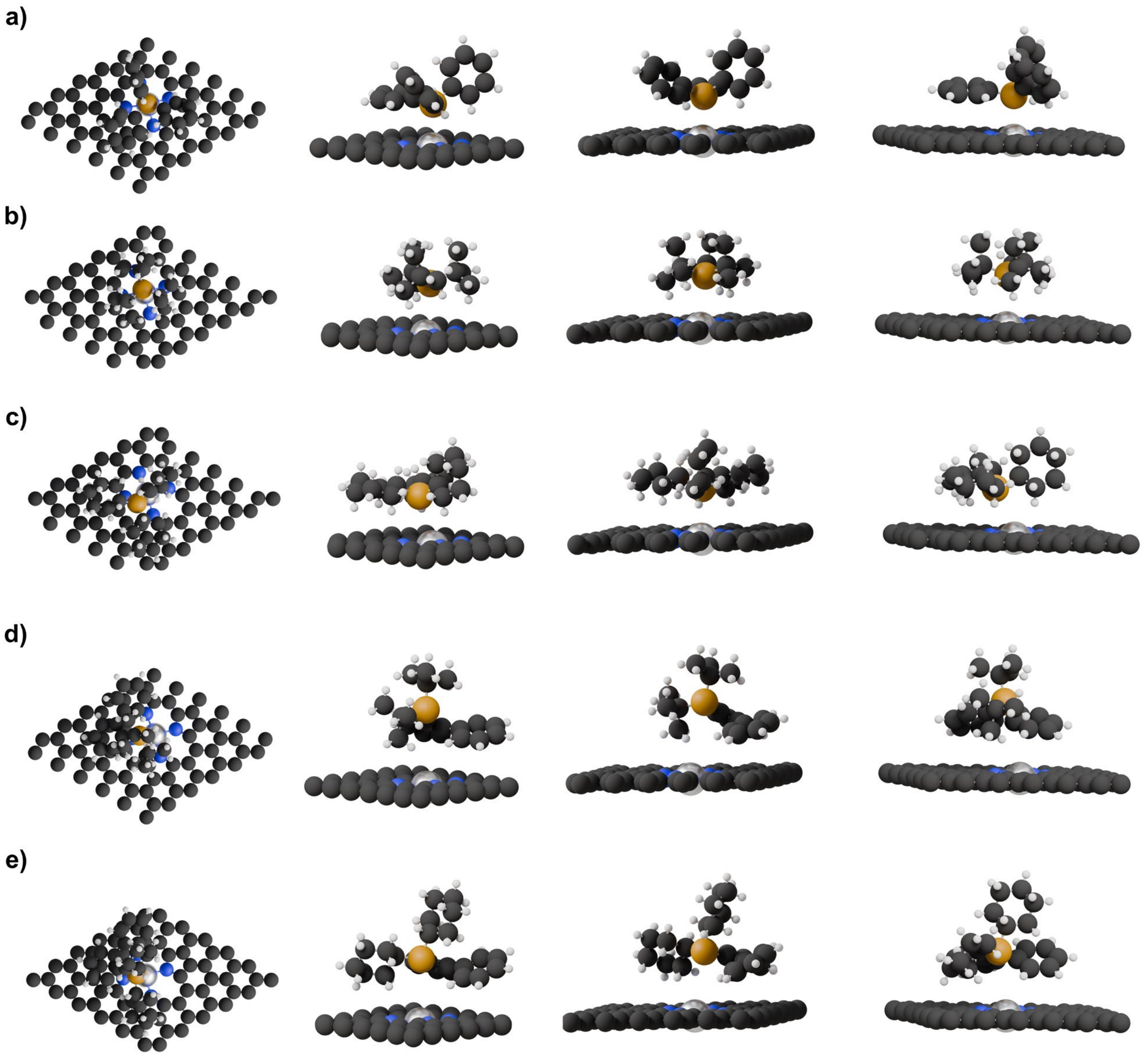

Figure S12. Most stable DFT-optimized phosphine–SAC configurations at the Pyr_{2+2} cavity for **a)** PPh_3, **b)** P^tBu_3, **c)** PCy_3, **d)** JP, and **e)** CJP (hydrogen: white, carbon: dark gray, nitrogen: blue, phosphorous: orange, palladium: silver). Based on the E_{ads} for this square planar in-plane configuration, which equals the adsorption of the phosphine on the GS, the palladium center is considered to remain inactive for interaction with the phosphine.

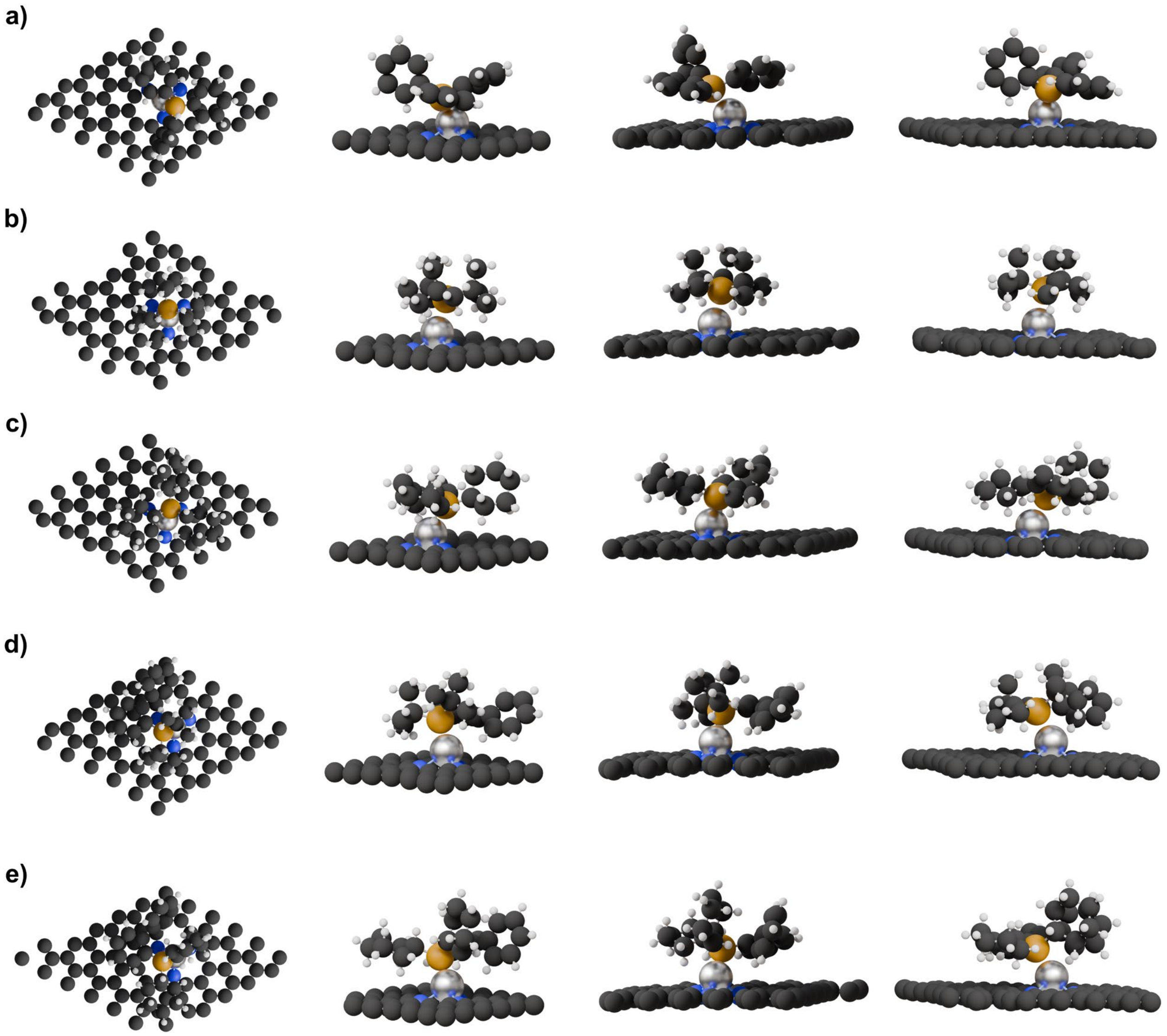

Figure S13. Most stable DFT-optimized phosphine–SAC configurations at the $Pyrr_3$ cavity for **a)** PPh_3, **b)** P^tBu_3, **c)** PCy_3, **d)** JP, and **e)** CJP (hydrogen: white, carbon: dark gray, nitrogen: blue, phosphorous: orange, palladium: silver). Based on the E_{ads} for this tetrahedral out-of-plane configuration, the palladium center engages in an interaction with the phosphine.

Topographic Heatmaps of Pd_{Pyrr3}–Phosphine Configurations

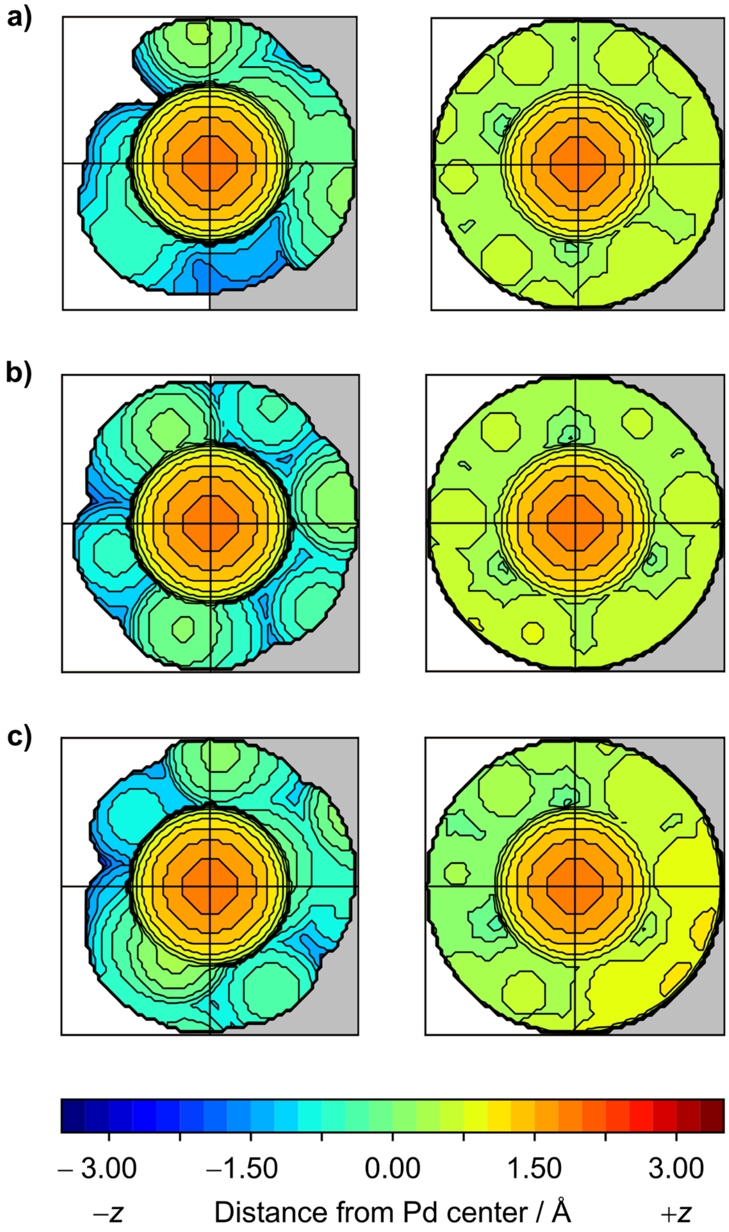

Figure S14. Topographic heatmaps visualizing the V_{bur} at the palladium atom. Pd_{Pyrr3}–phosphine (left column, ligand extends in the direction of $-z$) and Pd_{Pyrr3}–carrier (right column, carrier extends in the direction of $-z$) configurations for **a)** PPh_3, **b)** PCy_3 and **c)** P^tBu_3. Highlighted (white background) are the quadrants of the lowest steric demand. Positive values (red) of isocontour refer to the respective other hemisphere.

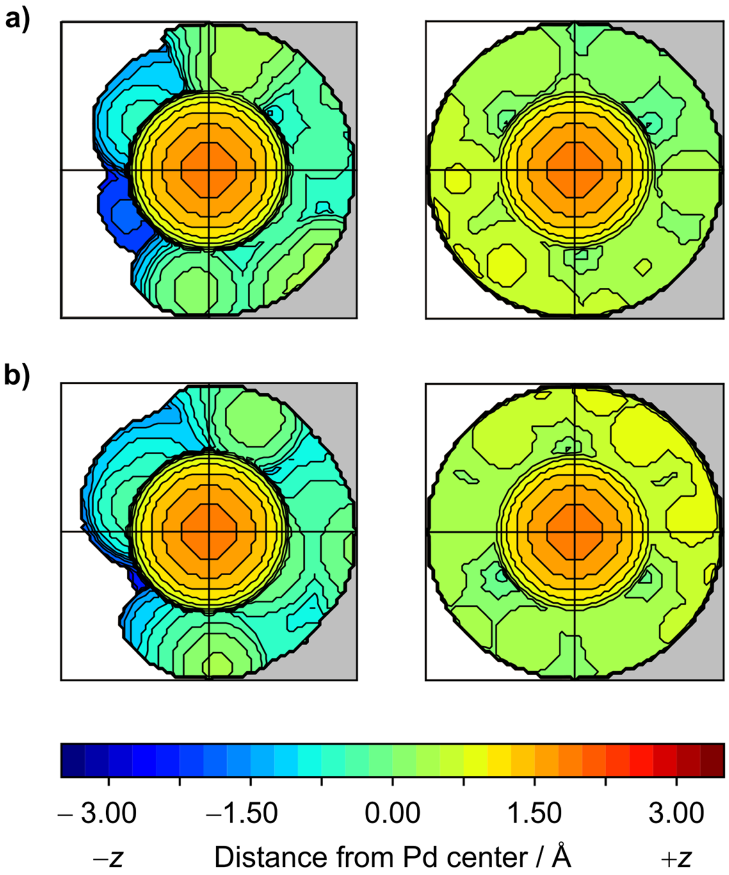

Figure S15. Topographic heatmaps visualizing the V_{bur} at the palladium atom. Pd_{Pyrr3}–phosphine (left column, ligand extends in the direction of –*z*) and Pd_{Pyrr3}–carrier (right column, carrier extends in the direction of –*z*) configurations for **a)** JohnPhos and **b)** CyJohnPhos. Highlighted (white background) are the quadrants of the lowest steric demand. Positive values (red) of isocontour refer to the respective other hemisphere.

Geometries of Iodobenzene–SAC–Phosphine Configurations

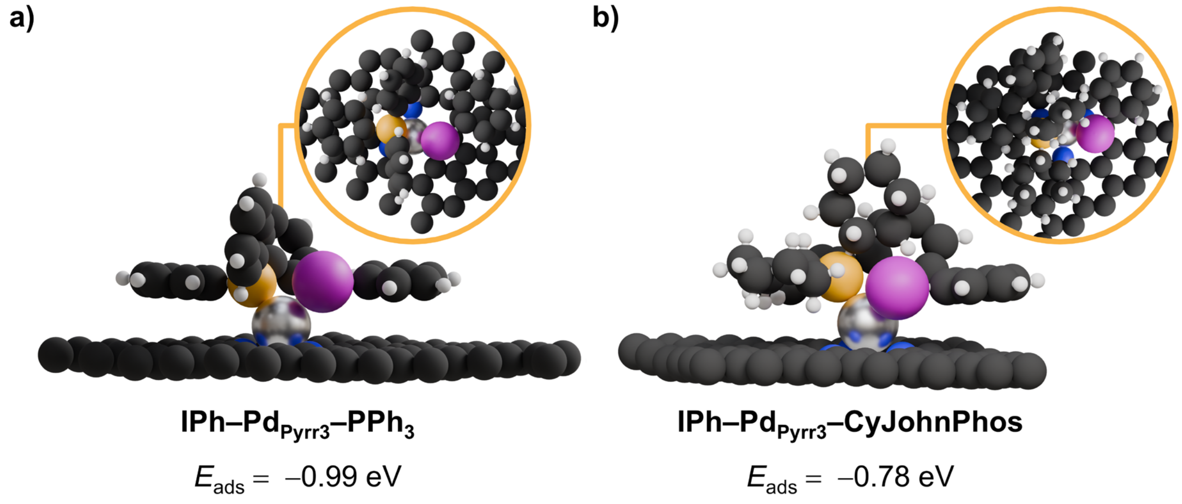

Figure S16. Most stable DFT-optimized IPh–SAC–phosphine configurations at the Pyrr$_3$ cavity for **a)** PPh$_3$ and **b)** CJP (hydrogen: white, carbon: dark gray, nitrogen: blue, phosphorous: orange, palladium: silver, iodine: purple) shown from the side and the top-down view (yellow circle).

Sonogashira-Hagihara Cross-Coupling Mechanism

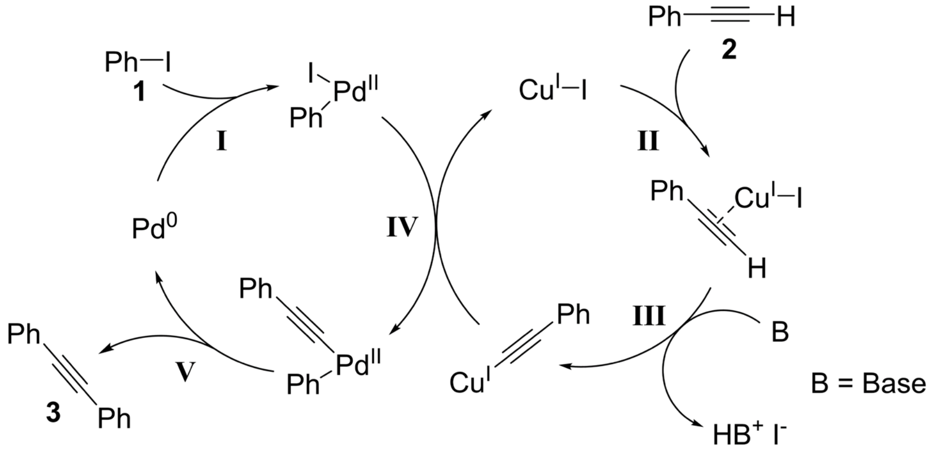

Scheme S1. Broadly accepted mechanism for the palladium-copper co-catalyzed Sonogashira-Hagihara coupling.[7] A palladium center in the Pd^0 oxidation state (either in the as-prepared catalyst or generated in situ) inserts into the aryl–halide bond during the oxidative addition (**I**), while the copper iodide activates the carbon–carbon triple bond for deprotonation through the base (**II**), generating a copper acetylide (**III**). Subsequently, the Pd^{II} and copper acetylide undergo transmetalation (**IV**) followed by reductive elimination of the final product (**V**).

References

(1) Dang, L. X. Mechanism and Thermodynamics of Ion Selectivity in Aqueous Solutions of 18-Crown-6 Ether: A Molecular Dynamics Study. *J. Am. Chem. Soc.* **1995**, *117* (26), 6954–6960. https://doi.org/10.1021/ja00131a018.

(2) Falivene, L.; Credendino, R.; Poater, A.; Petta, A.; Serra, L.; Oliva, R.; Scarano, V.; Cavallo, L. SambVca 2. A Web Tool for Analyzing Catalytic Pockets with Topographic Steric Maps. *Organometallics* **2016**, *35* (13), 2286–2293. https://doi.org/10.1021/acs.organomet.6b00371.

(3) Gensch, T.; dos Passos Gomes, G.; Friederich, P.; Peters, E.; Gaudin, T.; Pollice, R.; Jorner, K.; Nigam, A.; Lindner-D'Addario, M.; Sigman, M. S.; Aspuru-Guzik, A. A Comprehensive Discovery Platform for Organophosphorus Ligands for Catalysis. *J. Am. Chem. Soc.* **2022**, *144* (3), 1205–1217. https://doi.org/10.1021/jacs.1c09718.

(4) Newman-Stonebraker, S. H.; Smith, S. R.; Borowski, E.; Peters, E.; Gensch, T.; Johnson, H. C.; Sigman, M. S.; Doyle, A. G. Univariate Classification of Phosphine Ligation State and Reactivity in Cross-Coupling Catalysis. *Science* **2021**, *374* (6565), 301–308. https://doi.org/10.1126/science.abj4213.

(5) LeSueur, A.; Tao, N.; Doyle, A.; Sigman, M. Multi-Threshold Analysis for Chemical Space Mapping of Ni-Catalyzed Suzuki-Miyaura Couplings. *Eur. J. Org. Chem.* **2024**, *27* (36), 202400428. https://doi.org/10.1002/ejoc.202400428.

(6) Poier, D.; Akl, D. F.; Lucas, E.; Machado, A. R.; Giannakakis, G.; Mitchell, S.; Guillén-Gosálbez, G.; Marti, R.; Pérez-Ramírez, J. Reaction Environment Design for Multigram

Synthesis via Sonogashira Coupling over Heterogeneous Palladium Single-Atom Catalysts. *ACS Sustain. Chem. Eng.* **2023**, *11* (48), 16935–16945. https://doi.org/10.1021/acssuschemeng.3c04183.

(7) Chinchilla, R.; Nájera, C. The Sonogashira Reaction: A Booming Methodology in Synthetic Organic Chemistry. *Chem. Rev.* **2007**, *107* (3), 874–922. https://doi.org/10.1021/cr050992x.
